# Ultra-deep, long-read nanopore sequencing of mock microbial community standards

**DOI:** 10.1093/gigascience/giz043

**Published:** 2019-05-15

**Authors:** Samuel M Nicholls, Joshua C Quick, Shuiquan Tang, Nicholas J Loman

**Affiliations:** 1 Institute of Microbiology and Infection, School of Biosciences, University of Birmingham, Edgbaston, B15 2TT, UK; 2 Zymo Research Corporation, 17062 Murphy Ave., Irvine, CA 92614, USA

**Keywords:** bioinformatics, metagenomics, mock community, nanopore, single-molecule sequencing, real-time sequencing, benchmark, Illumina, *de novo* assembly

## Abstract

**Background:**

Long sequencing reads are information-rich: aiding *de novo* assembly and reference mapping, and consequently have great potential for the study of microbial communities. However, the best approaches for analysis of long-read metagenomic data are unknown. Additionally, rigorous evaluation of bioinformatics tools is hindered by a lack of long-read data from validated samples with known composition.

**Findings:**

We sequenced 2 commercially available mock communities containing 10 microbial species (ZymoBIOMICS Microbial Community Standards) with Oxford Nanopore GridION and PromethION. Both communities and the 10 individual species isolates were also sequenced with Illumina technology. We generated 14 and 16 gigabase pairs from 2 GridION flowcells and 150 and 153 gigabase pairs from 2 PromethION flowcells for the evenly distributed and log-distributed communities, respectively. Read length N50 ranged between 5.3 and 5.4 kilobase pairs over the 4 sequencing runs. Basecalls and corresponding signal data are made available (4.2 TB in total). Alignment to Illumina-sequenced isolates demonstrated the expected microbial species at anticipated abundances, with the limit of detection for the lowest abundance species below 50 cells (GridION). *De novo* assembly of metagenomes recovered long contiguous sequences without the need for pre-processing techniques such as binning.

**Conclusions:**

We present ultra-deep, long-read nanopore datasets from a well-defined mock community. These datasets will be useful for those developing bioinformatics methods for long-read metagenomics and for the validation and comparison of current laboratory and software pipelines.

## Data Description

Whole-genome sequencing of microbial communities (metagenomics) has revolutionized our view of microbial evolution and diversity, with numerous potential applications for microbial ecology, clinical microbiology, and industrial biotechnology [1,2]. Typically, metagenomic studies use high-throughput sequencing platforms (e.g., Illumina) [3], which generate very high yield, but of limited read length (100–300 base pairs [bp]).

In contrast, single-molecule sequencing platforms such as the Oxford Nanopore MinION, GridION, and PromethION are able to sequence very long fragments of DNA (>10 kilobase pairs [kb], with >2 megabase pairs [Mb] reported) [4,5], and with recent improvements to the platform making metagenomic studies using nanopore more viable, such studies are increasing in frequency [6–9]. Long reads help with alignment-based assignment of taxonomy and function owing to their increased information content [10,11]. Additionally, long reads permit bridging of repetitive sequences (within and between genomes), aiding genome completeness in *de novo* assembly [12]. However, these advantages are constrained by a high error rate (≈10%), necessitating the use of specific long-read alignment and assembly methods, which either are not specifically designed for metagenomics or have not been extensively tested on real data [13].

Mock community standards are useful for the development of genomics methods [14] and for the validation of existing laboratory, software, and bioinformatics approaches. For example, validating the accuracy of a taxonomic identification pipeline is important because the consequences of erroneous taxonomic identification from a metagenomic analysis may be severe, e.g., in public health microbiology [15,16] or incorrect diagnoses in clinical microbiology diagnostics. Mock community standards can also be used as positive controls during laboratory work, e.g., to validate that DNA extraction methods will yield the expected representation of a sampled community [14].

Here, we present 4 nanopore sequencing datasets of 2 microbial community standards, providing a state-of-the-art benchmark to accelerate the development of methods for analysing long-read metagenomics data.

### Background information

The ZymoBIOMICS Microbial Community Standards (CS and CSII) are each composed of 10 microbial species: 8 bacteria and 2 yeasts (Table 1). The organisms in CS (hereafter referred to as "Even") are distributed equally (12%), with the exception of the 2 yeasts, which are each present at 2%. Cell counts from organisms in the CSII ("Log") community are distributed on a log scale, ranging from 89.1% (*Listeria monocytogenes*) down to 0.000089% (*Staphylococcus aureus*).

**Table 1.** Description of the 10 organisms comprising the ZymoBIOMICS Mock Community Standards

Species	Type	Estimated size (Mb)	NRRL accession	ATCC accession	Sequence type	Illumina FASTQ	PacBio RSII FASTQ [17]	PacBio Sequel FASTQ [17]
*Bacillus subtilis*	Gram +	4.05	B-354	6633	ST7	ERR2935851	SRR7498042	SRR7415629
*Cryptococcus neoformans*	Yeast	18.90	Y-2534	32045		ERR2935856		
× *Cryptococcus deneoformans*								
*Enterococcus faecalis*	Gram +	2.85	B-537	7080	ST55	ERR2935850	SRR7415622	SRR7415630
*Escherichia coli*	Gram −	4.88	B-1109		ST10	ERR2935852	SRR7498041	
*Lactobacillus fermentum*	Gram +	1.91	B-1840	14931		ERR2935857		
*Listeria monocytogenes*	Gram +	2.99	B-33116	19117	ST449	ERR2935854	SRR7415624	SRR7415635
*Pseudomonas aeruginosa*	Gram −	6.79	B-3509	15442	ST252	ERR2935853	SRR7498043	
*Saccharomyces cerevisiae*	Yeast	12.10	Y-567	9763		ERR2935855	SRR7498048	SRR7415638
*Salmonella enterica*	Gram −	4.76	B-4212		ST139	ERR2935848	SRR7415626	SRR7415636
*Staphylococcus aureus*	Gram +	2.73	B-41012		ST9	ERR2935849	SRR7415627	SRR7415637

Table adapted from ZymoBIOMICS™ Microbial Community Standard II (Log Distribution) Instruction Manual v1.1.2 Table 2 and Appendix A. The *S. enterica* genome is listed at Agricultural Research Service Culture Collection (NRRL) (B-4212) as Serovar Typhimurium LT2, but our genomic analysis shows it is likely to be Serotype Choleraesuis, indicating possible mis-annotation. ATCC: American Type Culture Collection.

## Methods

### DNA extraction

DNA was extracted from 75 μl ZymoBIOMICS Microbial Community Standard (Zymo Research Corporation, Irvine, CA, USA. Product D6300, Lot ZRC190633) and 375 μl ZymoBIOMICS Microbial Community Standard II (Product D6310, Lot ZRC190842) using the ZymoBIOMICS DNA Miniprep extraction kit according to the manufacturer’s instructions, with the following modifications to increase fragment length and maintain the expected representation of the Gram-negative species that are already lysed in the DNA/RNA Shield storage solution. The standard was centrifuged at 8,000*×g* for 5 minutes before removing the supernatant and retaining. The cell pellet was resuspended in 750 μl lysis buffer and added to the ZR BashingBead lysis tube (Zymo Research Corporation). Bead-beating was performed on a FastPrep-24 (MP Biomedicals, Solon, OH, USA) instrument for 2 cycles of 40 seconds at 6.0 m s^−1^, with 5 minutes sitting on ice between cycles. The bead tubes were centrifuged at 10,000*×g* for 1 minute and 450 μl of supernatant was transferred to a Zymo Spin III-F filter before being centrifuged again at 8,000×*g*for 1 minute. 45 μl (Even) and 225 μl (Log) of the supernatant retained earlier was combined with 450 μl filtrate before adding 1485 μl (Even) or 2025 μl (Log) Binding Buffer and mixing before loading onto the column. Methods are available online via protocols.io [18].

### Nanopore sequencing library preparation

Quantification steps were performed using the dsDNA HS assay for Qubit. DNA was size-selected by cleaning up with 0.45× volume of Ampure XP (Beckman Coulter, Brea, CA, USA) and eluted in 100 μl EB (Qiagen, Hilden, Germany). Libraries were prepared from 1,400 ng input DNA using the SQK-LSK109 kit (Oxford Nanopore Technologies, Oxford, UK) in accordance with the manufacturer’s protocol, except incubation times for end repair, dA-tailing, and ligation were increased to 30 minutes to improve ligation efficiency. The Even and Log libraries were split and used on both the GridION and PromethION flowcells.

### Sequencing

Sequencing libraries were quantified and 2 aliquots of 50 and 400 ng were prepared for GridION and PromethION sequencing, respectively. The GridION sequencing was performed using FLO-MIN106 (rev.C) flowcells, MinKNOW 1.15.1, and standard 48-hour run script with active channel selection enabled. The PromethION sequencing was performed using FLO-PRO002 flowcells, MinKNOW 1.14.2, and standard 64-hour run script with active channel selection enabled.

Refuelling was performed approximately every 24 hours (GridION, PromethION) by loading 75 μl (GridION) or 150 μl (PromethION) refuelling mix (sequencing buffer diluted 1:1 with nuclease-free water). In addition, after the standard scripts had completed, the PromethION was restarted several times to utilize the remaining active pores and maximize total yield.

### Nanopore basecalling

Reads were basecalled on-instrument using the Guppy v2.2.2 GPU basecaller (Oxford Nanopore Technologies) with the supplied dna_r9.4.1_450bps_flipflop_prom.cfg configuration (PromethION) and dna_r9.4.1_450bps_flipflop.cfg (GridION).

### Illumina sequencing

DNA was extracted from pure cultures of each species using the ZymoBIOMICS DNA Miniprep Kit. Library preparation was performed using the Kapa HyperPlus Kit (Kapa Biosystems, Wilmington, MA, USA) with 100 ng DNA as input and TruSeq Y-adapters (Illumina, San Diego, CA, USA). The purified library derived from each sample was quantified with the 4200 TapeStation System (Agilent Technologies, Santa Clara, CA, USA) and pooled together in an equimolar fashion. The multiplexed isolates were sequenced on an Illumina HiSeq 1500 instrument using 2×101 bp (paired-end) sequencing, over 4 lanes. Raw reads were demultiplexed using bcl2fastq v2.17. Shotgun sequencing of the Even and Log communities was performed with the same protocol, with the exception that the Log community was sequenced individually on 2 flowcell lanes and the Even community was instead sequenced on an Illumina MiSeq using 2×151 bp (paired-end) sequencing.

## Bioinformatics Methods

### Illumina draft assembly

For the purposes of estimating sequencing coverage and contiguity, we constructed a draft assembly from our available Illumina sequencing data. Illumina reads for each of the 10 isolates were assembled using SPAdes v3.12.0 [19] with paired-end reads as input, using parameters -m 512 -t 12. Scaffolds from SPAdes <500 bp length or with <10× coverage were removed. The remaining scaffolds were combined into a single mock community draft assembly for downstream analysis. Multilocus sequence typing (MLST) of the scaffolds was conducted with mlst [20].

### Pacific Biosciences draft assembly

A recently released orthogonal data set from McIntyre et al. includes individual Pacific Biosciences (PacBio) sequencing of 8 of the 10 organisms that compose the 2 Zymo communities [17]. Assemblies for the 8 isolates that passed quality control (excluding *L. fermentum* and *C. neoformans*) were generated with HGAP v2 [21]. Assemblies have been made available by the authors and were downloaded from [22] (Git commit dba494d) for the purposes of assessing metagenomic assembly accuracy for the 7 bacterial species where complete genomes were available.

### Sequencing coverage estimation

Nanopore reads were aligned to the Illumina draft assembly using minimap2 [23] v2.14-r883 with parameters -ax map-ont -t 12 and converted to a sorted BAM file using samtools [24]. To reduce erroneous mappings, alignment BAM files were filtered using the script bamstats.py according to the following criteria: reference mapping length ≥500 bp, map quality (MAPQ) > 0, there are no supplementary alignments for this read, and read is not a secondary alignment. Per-species coverage summary statistics were generated using the summariseStats.R Rscript.

### Nanopore read accuracy

Read accuracy was determined by calculating BLAST-like identities from the filtered alignments (as per [25]), calculated as (*L* − *NM*)/*L* using the minimap2 number of mismatches (NM) SAM tag and the sum of match, insertion, and deletion CIGAR operations (L).

### Metagenomic assembly and contiguity estimation

Metagenomic assemblies were constructed with wtdbg2 v2.2 [26] from the nanopore sequencing of the communities. wtdbg2 was compiled from source via Git commit 904f2b3. For GridION, all nanopore reads were used. For PromethION, a 25% subsample was selected with seqtk [27].

Assemblies were conducted under a variety of parameter values for homopolymer-compressed k-mer size (-p), minimum graph edge weight support (-e), and read length threshold (-L). Global parameters for all runs (-S1 -K10000 -node-max 6000) were used to turn off k-mer subsampling (to remove assembly stochasticity) and increase the coverage thresholds applied to k-mers and constructed nodes.

Assembled contigs were assigned to taxa with kraken2 [28] (-use-names -t12) using a database containing all of the archaeal, bacterial, fungal, protozoal, and viral sequences from RefSeq, and UniVec_Core (database download links are in our repository). The kraken2 output was parsed with extracken.py and plotted with contiguity.R to visually assess contiguity. Following assignment, contigs can be extracted into separate FASTA with extract_contigs_with_kraken.py.

### Assembly polishing

After inspection of the contiguity.R plot, 8 high-contiguity assemblies were selected for polishing. Polishing consisted of 2 iterations of racon [29], followed by medaka [30] and 2 iterations of pilon [31]. racon v1.3.2 was used to polish contigs with the nanopore reads. medaka v0.5.0 was used to polish the racon polished contigs, with the nanopore reads specifying the r941_flip model. The PromethION assemblies were polished using the same seqtk-derived 25% subset from which the assemblies were constructed. pilon v1.23 was used to polish the medaka polished contigs, with the CS (Even) community Illumina reads.

### Estimation of genome completeness

To estimate accuracy of the polished assemblies, contigs were first assigned to taxa and extracted into separate FASTA using kraken2 as previously described. For the 7 bacteria for which corresponding PacBio draft assemblies were available, sequence identity dotplots were generated using a modified version of minidot [32], which uses minimap2 (-x asm10 -no-long-join -dual=yes -P) to align the polished contigs binned by kraken2, to the corresponding PacBio draft. Genome completeness was estimated with CheckM v1.0.13 [33] using the taxonomy_wf subcommand, after each phase of the polishing pipeline. CheckM was executed separately for each kraken2 bin that had a corresponding PacBio reference, specifying the appropriate species for the bin to taxonomy_wf. We report the CheckM “Completeness” score, which estimates completeness by identifying collocated marker gene sets on the assembled contigs as a proportion of the total collection of marker gene sets expected for a specific taxon.

## Results

### Nanopore sequencing metrics

**Figure 1:**
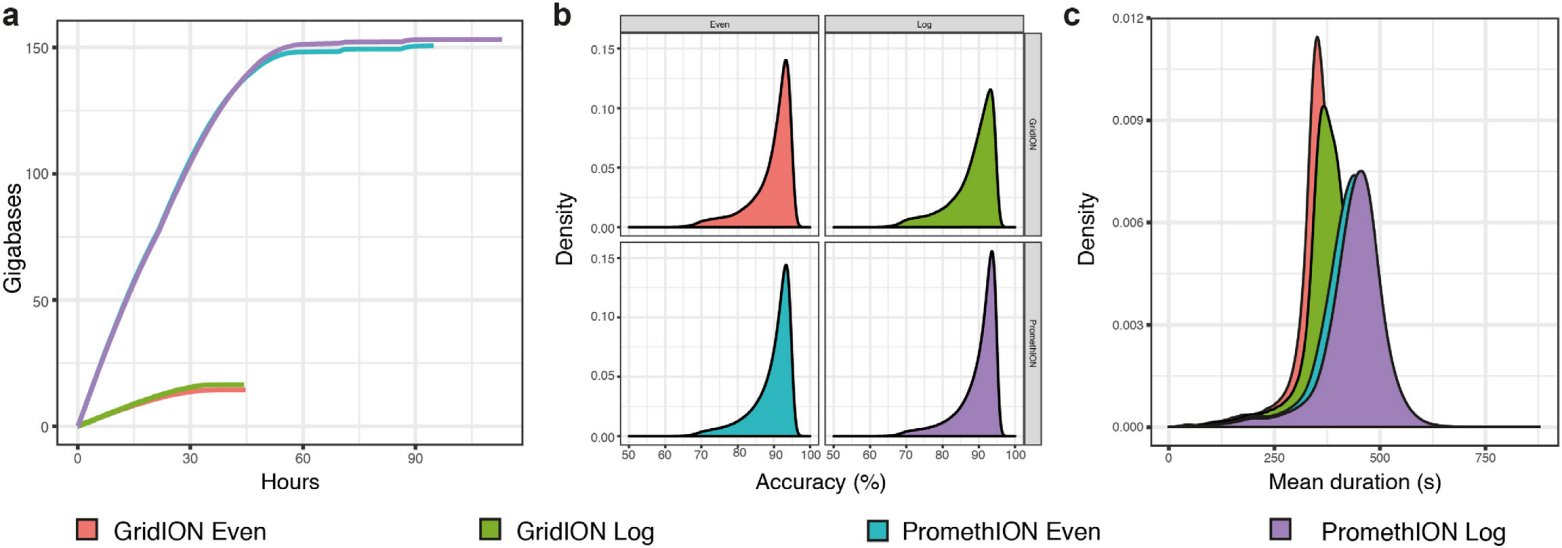
Summary plots for the 4 generated data sets: (**a**) collector’s curve showing sequencing yield over time for each of the 4 sequencing runs, (**b**) density plot showing sequence accuracy (BLAST-like identities), (**c**) density plot showing sequencing speed over time by sequencing experiment.

We generated a total of 335.1 gigabase pairs (Gb) of sequence from the 4 nanopore sequencing runs (Table 2, Fig. 1a). PromethION flowcells generated ∼10 times more sequencing data than the comparative GridION runs and showed equivalent read length N50 and read accuracy (Fig. 1b). We observed a difference in sequencing speed between the PromethION (mean speed, 419 and 437 bps for Even and Log, respectively) and the GridION (mean speed, 352 and 372 bp for Even and Log) (Fig. 1c).

**Table 2.** Summary of the 4 nanopore sequencing experiments

Signal accession	FASTQ accession	Sequencer	Standard (lot)	Time (h)	Reads (M)	N50 (kb)	Quality (median Q)	Yield (Gb)	Q>7 (Gb)
ERR2887847	ERR3152364	GridION	Zymo CS Even ZRC190633	48	3.49	5.3	10.3	14.38	12.39
ERR2887850	ERR3152366	GridION	Zymo CSII Log ZRC190842	48	3.67	5.4	9.8	16.51	13.97
ERR2887848	ERR3152365	PromethION	Zymo CS Even ZRC190633	64	35.7	5.4	10.5	150.88	130.32
ERR2887849	PromethION	Zymo CS Even ZRC190633							
ERR2887851	ERR3152367	PromethION	Zymo CSII Log ZRC190842	64	34.5	5.4	10.7	153.31	133.68
ERR2887852	PromethION	Zymo CSII Log ZRC190842							

PromethION runs were restarted following the standard 64-hour protocol. The table reflects total yield across both the standard run and subsequent restarts.

### Illumina sequencing metrics

Illumina datasets for the 10 individually sequenced isolates averaged 13.53 million pairs of reads (ranging between 7.1 and 23.2 million), with proportions of reads with a mean phred score ≥30 ranging between 75.51% and 93.09% (Table 3). Illumina sequencing generated 8.8 million pairs of reads (2×151 bp, MiSeq) and 47.8 million pairs of reads (2×101 bp, HiSeq) for the Even and Log community, respectively (Table 3).

**Table 3.** Summary statistics for Illumina sequencing data

Dataset	Pairs (M)	Yield (Gb)	phred ≥ 30 (%)	Accession
Isolates	13.53 ± 5.23	2.73 ± 1.06	87.72 ± 5.43	See Table 1
CS (Even)	8.8	2.65	95.12	ERR2984773
CSII (Log)	47.8	9.66	95.71	ERR2935805

Illumina sequencing was performed on an Illumina HiSeq 1500, with the exception of the Even community, which was sequenced on an Illumina MiSeq.

### Nanopore mapping statistics

**Figure 2:**
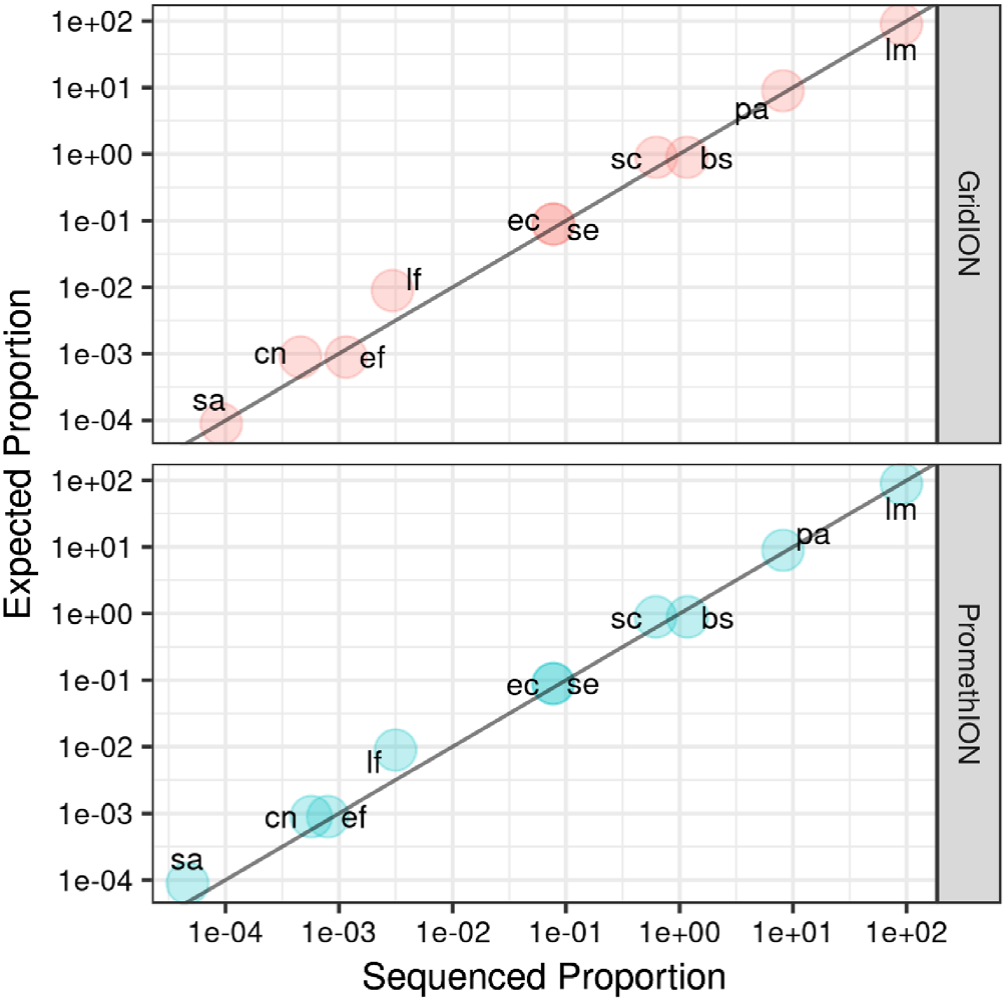
Proportion of sequenced bases assigned by minimap2 to each of the 10 organisms that were sequenced (x-axis), against the proportion of yield expected given the known composition (y-axis) of the Zymo CSII (Log) standard.

We identify the presence of all 10 microbial species in the community, for both Even and Log samples, in expected proportions (Fig. 2). For the Even community, the GridION results provide sufficient depth (i.e., ≫30× coverage) to potentially assemble all 8 of the bacteria. The coverage of the yeast genomes was lower (10× and 17×), potentially sufficient for assembly scaffolding. On the PromethION all genomes had >100× mean coverage (Tables 4 and 5).

**Table 4.** Read alignment statistics for Even samples, showing absolute measurements and proportion of sequencing yield and the estimated genome coverage obtained for each organism in the mock community

		GridION				PromethION			
Species	Expected proportion	Yield (Gb)	Measured proportion	Alignment N50 (kb)	Coverage (×)	Yield (Gb)	Measured proportion	Alignment N50 (kb)	Coverage (×)
*Bacillus subtilis*	12	2.12	19.32	4.30	524.51	21.55	19.02	4.40	5,326.44
*Listeria monocytogenes*	12	1.60	14.56	4.47	534.26	16.23	14.33	4.58	5,424.46
*Enterococcus faecalis*	12	1.34	12.24	4.45	472.47	13.67	12.07	4.57	4,805.60
*Staphylococcus aureus*	12	1.24	11.28	4.47	453.84	12.59	11.11	4.59	4,611.61
*Salmonella enterica*	12	1.10	9.99	8.55	230.51	11.69	10.32	8.95	2,456.19
*Escherichia coli*	12	1.09	9.93	8.31	223.59	11.62	10.26	8.71	2,382.59
*Pseudomonas aeruginosa*	12	1.07	9.70	8.98	156.85	11.45	10.11	9.38	1,686.34
*Lactobacillus fermentum*	12	1.02	9.28	3.62	534.73	10.34	9.13	3.73	5,425.69
*Saccharomyces cerevisiae*	2	0.21	1.92	4.09	17.46	2.12	1.87	4.18	175.23
*Cryptococcus neoformans*	2	0.20	1.78	4.45	10.37	2.00	1.77	4.54	105.82

**Table 5.** Read alignment statistics for Log samples, describing sequencing yield and estimated genome coverage obtained for each organism in the mock community

	GridION			PromethION		
Species	Yield (Gb)	Alignment N50 (kb)	Coverage (×)	Yield (Gb)	Alignment N50 (kb)	Coverage (×)
*Listeria monocytogenes*	12.10	4.95	4,043.90	110.09	4.97	36,796.21
*Pseudomonas aeruginosa*	1.10	9.38	161.45	9.99	9.33	1,471.41
*Bacillus subtilis*	0.16	5.03	38.67	1.44	5.04	356.00
*Saccharomyces cerevisiae*	0.08	4.78	6.93	0.75	4.75	62.33
*Salmonella enterica*	0.01	9.20	2.20	0.10	9.17	20.04
*Escherichia coli*	0.01	8.65	2.14	0.09	9.17	19.24
*Lactobacillus fermentum*	4E−4	3.40	0.210	0.004	3.37	2.03
*Enterococcus faecalis*	2E−4	7.62	0.055	1E−3	6.05	0.34
*Cryptococcus neoformans*	6E−5	4.41	0.003	7E−4	4.97	0.037
*Staphylococcus aureus*	1E−5	7.12	0.005	5E−5	3.58	0.020

Note that expected and measured proportions are illustrated in Fig. 2.

For the log-distributed community, 3 taxa have sufficient coverage for assembly on GridION, compared with 4 on PromethION. On PromethION, a further 2 genomes (*S. enterica* and *E. coli*) have sufficient coverage for assembly scaffolding. We were able to detect *S. aureus*, the lowest abundance organism on both platforms, with 19 reads from PromethION (from 400-cell input) and 4 reads from GridION (from 50-cell input).

### Nanopore metagenomic assemblies

We assessed the contiguity of our nanopore metagenomic assemblies for each run with different assembly parameters.

**Figure 3:**
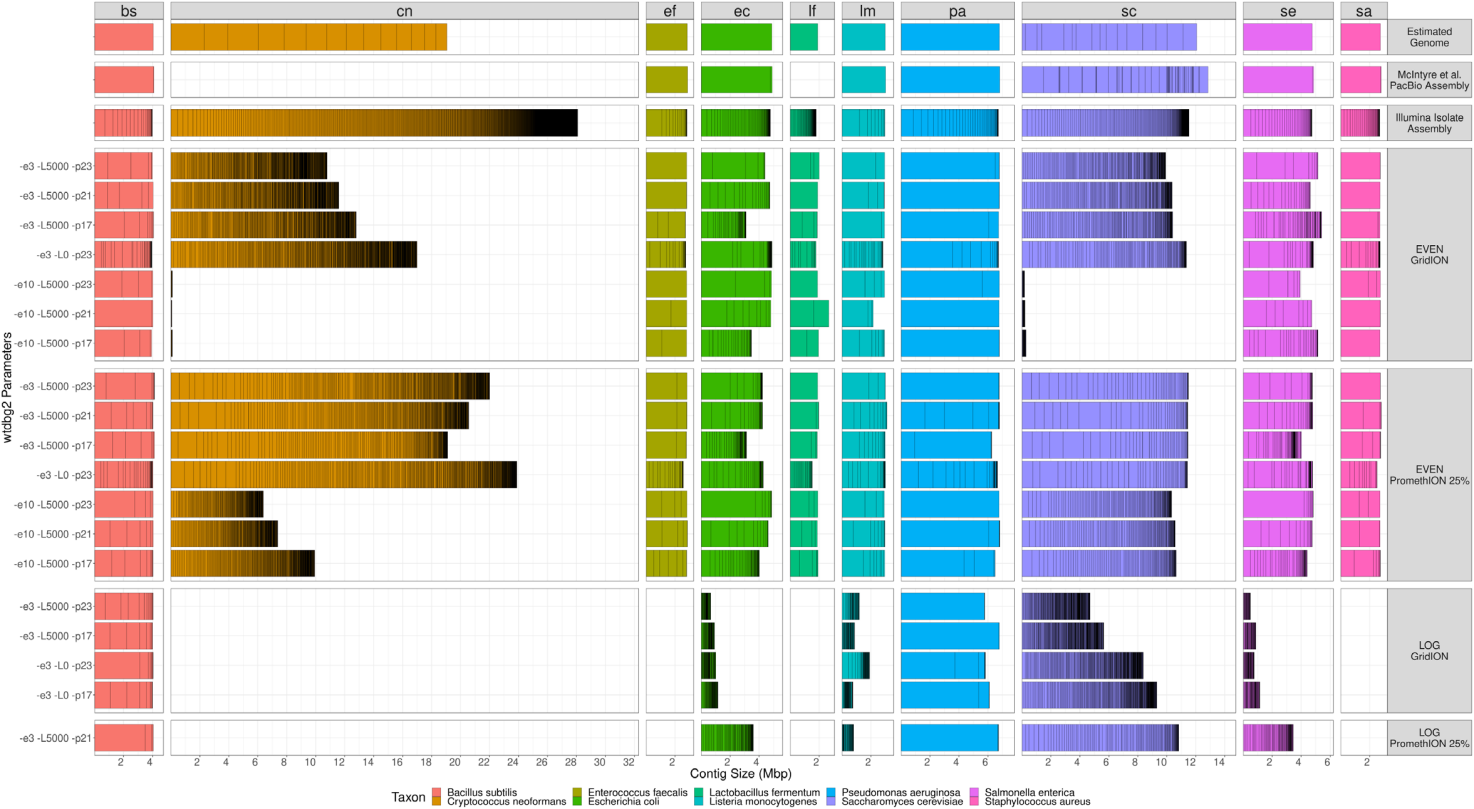
Bar plots demonstrating total length and contiguity of genomic assemblies obtained with wtdbg2 from each of the long-read nanopore data sets. For each organism in the community (coloured columns), contigs longer than 10 kb are horizontally stacked along the x-axis. Each row represents a run of wtdbg2, with the parameters for edge support, read length threshold, and homopolymer-compressed k-mer size labelled on the left. Assemblies are grouped by the data set on which they were run (row facets). Additionally, assemblies may be compared to the estimated true genome size, the available McIntyre et al. [17] PacBio assemblies, and per-isolate Illumina SPAdes assembly. Estimated genomes sizes are the same as those found in Table 1; however, to display approximate chromosomes, the 2 yeasts were replaced by their corresponding canonical National Center for Biotechnology Information references for visualization purposes only. The *C. neoformans* strain used by the Zymo standards is a diploid genetic cross, which may explain the larger assemblies, compared to the represented estimated haploid size.

For the Even community, genomes of the expected size were present for each of the bacterial species, contained in small numbers of large contigs (Fig. 3). However, the 2 yeasts are highly fragmented, consistent with their low read depth.

*L. monocytogenes* is poorly assembled in the Log dataset despite being the most abundant organism, indicating that very high sequence coverage may be detrimental to the performance of wtdbg2. We note that assembling the entire PromethION dataset resulted in less complete and more fragmented assemblies. This led us to random subsample the PromethION data to 25% of the total dataset, which improved the assembly results.

After subsampling, assemblies of the Even community from GridION and PromethION were similar. However, the assemblies from PromethION data had better representation of the yeasts in terms of size and contiguity (particularly for *C. neoformans*), likely due to the higher coverage of these species.

We also assessed the completeness of polished genomes for a selection of our highly contiguous metagenomic assemblies.

For GridION, we observed that for ≥1 of the polished assemblies, 4 bacterial genomes are reconstructed to ≥95% of their length (L95) in a single contig. For PromethION, we observed that for 7 bacteria, at least half the genome (L50) is reconstructed on a single contig, for ≥1 assembly condition (Table 6).

**Table 6.**
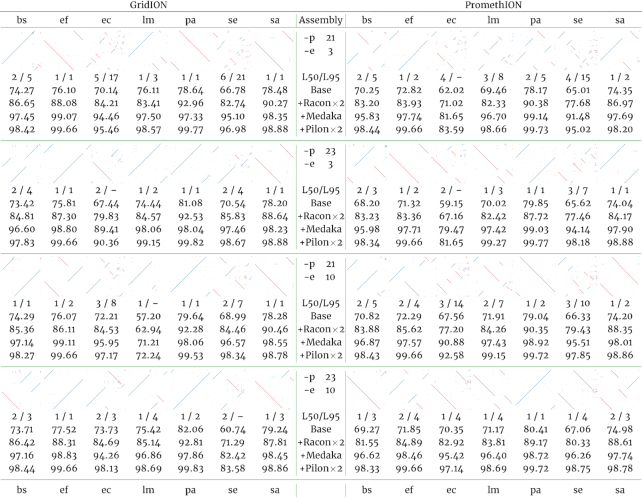
Sequence identity dotplots and CheckM genome completeness scores for each of the 7 bacterial species for which there was a corresponding PacBio assembly from McIntyre et al. [17]

Four wtdbg2 assembly conditions are represented, varying the homopolymer-compressed k-mer parameter "p" and the graph minimum edge weight threshold “e.” The read length threshold “L” was fixed at 5,000 bp. The left and right halves of the table correspond to the same assembly condition for the GridION and 25% PromethION sequencing data, respectively. The L50/L95 refers to the number of assembled contigs required to span ≥50% and ≥95% of the estimated genome size (see Table 1). A minus sign indicates that the set of assembled contigs assigned to a taxon were not of sufficient total length to cover 95% of the estimated size. CheckM genome completeness scores are expressed as a percentage and were calculated per organism at the end of each polishing phase. bs: *B. subtilis*; ef: *E. faecalis*; ec: *E. coli*; lm: *L. monocytogenes*; pa: *P. aeruginosa*; se: *S. enterica*; sa: *S. aureus*.

Genome completeness as estimated by CheckM averaged 73.95% and 70.98% over the 4 unpolished assemblies, for the GridION and PromethION assemblies, respectively. We observed that each phase of the polishing pipeline improved completeness. For the GridION assemblies, completeness was incrementally improved by 11.57, 10.14, and 1.25 percentage points for 2 iterations of racon, 1 iteration of medaka, and 2 iterations of short-read polishing with pilon, respectively. For the PromethION assemblies, the 3 polishing phases incrementally improved assemblies by an average of 11.92, 12.69, and 1.77 percentage points. In almost all cases, polishing yielded near-complete (≥90%) genomes.

## Discussion

There are several noteworthy aspects of this dataset: we generated >300 Gb of sequence data from the Oxford Nanopore PromethION and 30 Gb from the Oxford Nanopore GridION, on a well-characterized mock community sample, and we have made basecalls and electrical signal data for each of the 4 runs presented here available: a combined dataset size of >4 terabytes. The availability of the raw signal permits future basecalling of the data (an area under rapid development), as well as signal-level polishing and the detection of methylated bases [34].

Individual sequencing libraries were split between the GridION and PromethION, permitting direct comparisons of the instruments to be made. We observed high concordance between the datasets from each platform. We note that the sequencing speed of the PromethION is faster than the GridION, which we attribute to different running temperatures on these instruments (39°C vs 34°C, respectively).

Confident detection of *S. aureus* was demonstrated for the GridION run to <50 cells using the Log community. The PromethION generated ∼5 times more *S. aureus* reads than the GridION; however, we loaded 8 times as much library, making it seem less sensitive. It may be possible to reduce the input to PromethION flowcells, but we have not attempted this.

Early results of metagenomic assembly show promise for reconstruction of whole microbial genomes from mixed samples without a binning step. We focused on the developing wtdbg2 software because the established minimap2 and miniasm method resulted in excessively large intermediate files (tens of terabases per analysis) that were impractical to store and analyse.

For the Even community, using wtdgb2 with varying parameter choices, we were able to assemble 4 of the bacteria into single contigs. However, no single parameter set was found to be optimum for both total genome size and contig length. Increasing -e improved contiguity for the Even community; however, this resulted in the loss of yeasts from the assembly. Increasing the read length threshold (-L) improved contiguity for all sample and platform combinations, at the cost of genome size. Increasing the homopolymer-compressed k-mer size (-p) from the default of 21 to 23 also seemed to improve contiguity.

We found that wtdbg2 expects a maximum of 200× sample coverage and discards sequence k-mers and de Bruijn graph nodes with >200× support. Although these limits can be lifted by specifying higher -K and –node-max, we still observe more fragmented assemblies on the PromethION data (especially for the 100% PromethION data [not shown]), potentially indicating a need to further tune the algorithm to account for the large differences in coverage between genomes. It should be noted that wtdbg2 is still under active development, making it difficult to make concrete recommendations for parameters.

We found that any form of polishing improves the completeness of assemblies, likely due to the correction of frameshifts caused by indels. Short-read polishing with pilon also improves the assemblies, despite low coverage of the Illumina Even community data, and the results might be expected to improve further with increased coverage.

The availability of this dataset should help with further improvements to long-read assembly techniques.

Other mock microbial samples are available that we did not test here. A notable alternative mock community sample is from the Human Microbiome Project (HMP) and consists of 20 microbial samples (available from BEI Resources). This mock community have been sequenced as part of other studies, although the datasets are much smaller than the ones presented here [9,35]. Bertrand et al. [12] presented a synthetic mock community of their own construction to demonstrate hybrid nanopore-Illumina metagenome assemblies.

### Re-use potential

The provision of Illumina reads for each isolate permits a ground-truth to be obtained for the individual species contained in the mock community. This will be useful for training new nanopore basecalling and polishing models, long-read aligners, variant callers, and validating taxonomic assignment and assembly software and pipelines.

## Availability of source code and requirements

Python and R scripts used to generate the summary information and analyses are open source and freely available via our repository (https://github.com/LomanLab/mockcommunity), under the MIT license. Our pipeline was orchestrated with Snakemake [36]; the workflow is available from our repository.

## Availability of supporting data and materials

This manuscript, and its supporting data are available under a Creative Commons Attribution 4.0 International license.

Unprocessed FASTQ from the Illumina sequencing of the 10 isolates is available at the European Nucleotide Archive, via the identifiers listed in Table 1; identifiers for the Even and Log community Illumina sequencing can be found in Table 3.

Both the raw signal, and basecalled FASTQ for our nanopore sequencing experiments are available at the European Nucleotide Archive, via the identifiers listed in Table 2.

The SPAdes-assembled Illumina draft reference, and the collection of nanopore assemblies for each wtdbg2 condition are linked to from our GitHub repository (https://github.com/LomanLab/mockcommunity), along with the kraken2 database used for taxonomic classification of the assembled contigs.

Further updates (such as updated references, or new assemblies) will be made available through our project website https://lomanlab.github.io/mockcommunity/.

An archival snapshot of our GitHub repository and associated assembly FASTA files are also available via GigaDB [37].

## Abbreviations

ATCC: American Type Culture Collection; bp: base pairs; CS: Community Standards; Gb: gigabase pairs; kb: kilobase pairs; Mb: megabase pairs; MLST: multilocus sequence typing; NRRL: Northern Regional Research Laboratory; ARSCC: Agricultural Research Service Culture Collection; PacBio: Pacific Biosciences.

## Competing interests

Cambridge Biosciences provided ZymoBIOMICS products free of charge. S.T. is an employee of Zymo Research Corporation. N.J. has received Oxford Nanopore Technologies (ONT) reagents free of charge to support his research programme. N.J. and J.Q. have received travel expenses to speak at ONT events. N.L. has received an honorarium to speak at an ONT company meeting.

## Funding

S.N. is funded by the Medical Research Foundation and the National Institute for Health Research (NIHR) STOP-COLITIS project. J.Q. is funded by the NIHR Surgical Reconstruction and Microbiology Research Centre, which is a partnership between the NIHR, University Hospitals Birmingham NHS Foundation Trust, the University of Birmingham, and the Royal Centre for Defence Medicine. N.L. is funded by an MRC Fellowship in Microbial Bioinformatics under the CLIMB project.

## Authors' contributions

Conceptualization: N.L.; Methodology: N.L., J.Q., S.N., S.T.; Software: S.N., N.L.; Validation: S.N., N.L.; Formal analysis: S.N., N.L.; Investigation: N.L., J.Q., S.N.; Resources: N.L., S.T.; Data curation: S.N., N.L., S.T.; Writing—original draft preparation: S.N.; Writing—review and editing: S.N., N.L., J.Q., S.T.; Visualization: S.N., N.L.; Supervision: N.L.; Project administration: N.L.; Funding acquisition: N.L., S.T.

## Supplementary Material

GIGA-D-18-00495_Original_Submission.pdf

GIGA-D-18-00495_Revision_1.pdf

GIGA-D-18-00495_Revision_2.pdf

Response_to_Reviewer_Comments_Original_Submission.pdf

Response_to_Reviewer_Comments_Revision_1.pdf

Reviewer_1_Report_Original_Submission -- Lachlan Coin 12/29/2018 Reviewed

Reviewer_1_Report_Revision_1 -- Lachlan Coin 3/4/2019 Reviewed

Reviewer_2_Report_Original_Submission -- Ameet Pinto, PhD 1/3/2019 Reviewed
