# Supplementary material for: Ultra-deep, long-read nanopore sequencing of mock microbial community standards
Source: Gigascience. 2019 May 15;8(5):giz043. doi: 10.1093/gigascience/giz043 (PMC6520541; doi:10.1093/gigascience/giz043)
Supplement: GIGA-D-18-00495_Original_Submission.pdf [file giz043_giga-d-18-00495_original_submission.pdf]

GigaScience

Ultra-deep, long-read nanopore sequencing of mock microbial community standards

--Manuscript Draft--

**Manuscript Number:**	GIGA-D-18-00495
**Full Title:**	Ultra-deep, long-read nanopore sequencing of mock microbial community standards
**Article Type:**	Data Note
**Funding Information:**	
**Abstract:**	Background: Long sequencing reads are information-rich: aiding de novo assembly and reference mapping, and consequently have great potential for the study of microbial communities. However, the best approaches for analysis of long-read metagenomic data are unknown. Additionally, rigorous evaluation of bioinformatics tools is hindered by a lack of long-read data from validated samples with known composition.<br>Methods: We sequenced two commercially-available mock communities containing ten microbial species (ZymoBIOMICS Microbial Community Standards) with Oxford Nanopore GridION and PromethION. Isolates from the same mock community were sequenced individually with Illumina HiSeq.<br>Data: We generated 14 and 16 Gbp from GridION flowcells and 146 and 148 Gbp from PromethION flowcells for the even and odd communities respectively. Read length N50 was 5.3 Kbp and 5.2 Kbp for the even and log community, respectively. Basecalls and corresponding signal data are made available (4.2 TB in total).<br>Results: Alignment to Illumina-sequenced isolates demonstrated the expected microbial species at anticipated abundances, with the limit of detection for the lowest abundance species below 50 cells (GridION). De novo assembly of metagenomes recovered long contiguous sequences without the need for pre-processing techniques such as binning.<br>Conclusions: We present ultra-deep, long-read nanopore datasets from a well-defined mock community. These datasets will be useful for those developing bioinformatics methods for long-read metagenomics and for the validation and comparison of current laboratory and software pipelines.
**Corresponding Author:**	Nick Loman<br><br>UNITED KINGDOM
**Corresponding Author Secondary Information:**	
**Corresponding Author's Institution:**	
**Corresponding Author's Secondary Institution:**	
**First Author:**	Samuel M Nicholls
**First Author Secondary Information:**	
**Order of Authors:**	Samuel M Nicholls
	Joshua C Quick
	Shuiquan Tang
	Nick Loman
**Order of Authors Secondary Information:**	
**Additional Information:**	
**Question**	**Response**
Are you submitting this manuscript to a special series or article collection?	No

Powered by Editorial Manager® and ProduXion Manager® from Aries Systems Corporation

**Experimental design and statistics**<br><br>Full details of the experimental design and statistical methods used should be given in the Methods section, as detailed in our Minimum Standards Reporting Checklist. Information essential to interpreting the data presented should be made available in the figure legends.<br><br>Have you included all the information requested in your manuscript?	Yes
**Resources**<br><br>A description of all resources used, including antibodies, cell lines, animals and software tools, with enough information to allow them to be uniquely identified, should be included in the Methods section. Authors are strongly encouraged to cite Research Resource Identifiers (RRIDs) for antibodies, model organisms and tools, where possible.<br><br>Have you included the information requested as detailed in our Minimum Standards Reporting Checklist?	Yes
**Availability of data and materials**<br><br>All datasets and code on which the conclusions of the paper rely must be either included in your submission or deposited in publicly available repositories (where available and ethically appropriate), referencing such data using a unique identifier in the references and in the “Availability of Data and Materials” section of your manuscript.<br><br>Have you have met the above requirement as detailed in our Minimum Standards Reporting Checklist?	Yes

Powered by Editorial Manager® and ProduXion Manager® from Aries Systems Corporation

Manuscript
Click here to access/download;Manuscript;msfile-1.pdf
Click here to view linked References

bioR$_\chi$iv, 2018, 1–7

doi: xx.xxxx/xxxx
Manuscript in Preparation
Data Note

DATA NOTE

Ultra-deep, long-read nanopore sequencing of mock microbial community standards

Samuel M. Nicholls[1†], Joshua C. Quick[1†], Shuiquan Tang[2] and Nicholas J. Loman[1*]

[1]Institute of Microbiology and Infection, School of Biosciences, University of Birmingham, UK and [2]Zymo Research Corporation, Irvine, California, USA

[*]n.j.loman@bham.ac.uk
[†]Contributed equally.

Abstract

Background: Long sequencing reads are information-rich: aiding *de novo* assembly and reference mapping, and consequently have great potential for the study of microbial communities. However, the best approaches for analysis of long-read metagenomic data are unknown. Additionally, rigorous evaluation of bioinformatics tools is hindered by a lack of long-read data from validated samples with known composition.
Methods: We sequenced two commercially-available mock communities containing ten microbial species (ZymoBIOMICS Microbial Community Standards) with Oxford Nanopore GridION and PromethION. Both communities and the ten individual species isolates were also sequenced with Illumina technology.
Data: We generated 14 and 16 Gbp from GridION flowcells and 146 and 148 Gbp from PromethION flowcells for the even and log communities respectively. Read length N50 was 5.3 Kbp and 5.2 Kbp for the even and log community, respectively. Basecalls and corresponding signal data are made available (4.2 TB in total).
Results: Alignment to Illumina-sequenced isolates demonstrated the expected microbial species at anticipated abundances, with the limit of detection for the lowest abundance species below 50 cells (GridION). *De novo* assembly of metagenomes recovered long contiguous sequences without the need for pre-processing techniques such as binning.
Conclusions: We present ultra-deep, long-read nanopore datasets from a well-defined mock community. These datasets will be useful for those developing bioinformatics methods for long-read metagenomics and for the validation and comparison of current laboratory and software pipelines.

Key words: bioinformatics; metagenomics; mock community; nanopore; single-molecule sequencing; real-time sequencing; benchmark; GridION; PromethION; Illumina; *de novo* assembly

Data Description

Whole-genome sequencing of microbial communities (metagenomics) has revolutionised our view of microbial evolution and diversity, with numerous potential applications for microbial ecology, clinical microbiology and industrial biotechnology [1, 2]. Typically, metagenomic studies use high-throughput sequencing platforms (*e.g.* Illumina) [3] which generate very high yield, but of limited read length (100–300 bp).

In contrast, single-molecule sequencing platforms such as the Oxford Nanopore MinION, GridION and PromethION are able to sequence very long fragments of DNA (>10 Kbp, with over 2 Mbp reported) [4, 5] and with recent improvements to the platform making metagenomic studies using nanopore more viable, such studies are increasing in frequency [6, 7, 8, 9]. Long reads help with alignment-based assignment of taxonomy and function due to their increased information content [10, 11]. Additionally, long reads permit bridg-

Compiled on: December 10, 2018.
Draft manuscript prepared by the author.

Table 1. Description of the ten organisms comprising the ZymoBIOMICS Mock Community Standards.

Species	Type	Estimated Size (Mbp)	NRRL Accession	ATCC Accession	Sequence Type	Illumina FASTQ
Bacillus subtilis	Gram +	4.045	B-354	6633	ST7	ERR2935851
Cryptococcus neoformans × *Cryptococcus deneoformans*	Yeast	18.9	Y-2534	–	–	ERR2935856
Enterococcus faecalis	Gram +	2.845	B-537	7080	ST55	ERR2935850
Escherichia coli	Gram –	4.875	B-1109	–	ST10	ERR2935852
Lactobacillus fermentum	Gram +	1.905	B-1840	14931	–	ERR2935857
Listeria monocytogenes	Gram +	2.992	B-33116	19117	ST449	ERR2935854
Pseudomonas aeruginosa	Gram –	6.792	B-3509	15442	ST252	ERR2935853
Saccharomyces cerevisiae	Yeast	12.1	Y-567	9763	–	ERR2935855
Salmonella enterica	Gram –	4.760	B-4212	–	ST139	ERR2935848
Staphylococcus aureus	Gram +	2.730	B-41012	–	ST9	ERR2935849

Table adapted from ZymoBIOMICS™ Microbial Community Standard II (Log Distribution) Instruction Manual v1.1.2 Table 2 and Appendix A. The *S. enterica* genome is listed at NRRL (B-4212) as Serovar Typhimurium LT2, but our genomic analysis shows it is likely to be Serotype Choleraesuis; indicating possible mis-annotation.

ing of repetitive sequences (within and between genomes), aiding genome completeness in *de novo* assembly [12]. However, these advantages are constrained by high error rate (≈10%), requiring the use of specific long-read alignment and assembly methods, which are either not specifically designed for metagenomics, or have not been extensively tested on real data [13].

Mock community standards are useful for the development of genomics methods [14], and for the validation of existing laboratory, software and bioinformatics approaches. For example, validating the accuracy of a taxonomic identification pipeline is important, because the consequences of erroneous taxonomic identification from a metagenomic analysis may be severe, *e.g.* in public health microbiology (such as in the well-publicised case of anthrax and plague in the New York Subway [15]) or missed diagnoses in the clinic. Mock community standards can also be used as positive controls during laboratory work, for example to validate that DNA extraction methods will yield expected representation of a sampled community [14].

Here, we present four nanopore sequencing datasets of two microbial community standards, providing a state-of-the-art benchmark to accelerate the development of methods for analysing long-read metagenomics data.

Background Information

The ZymoBIOMICS Microbial Community Standards (CS and CSII) are each composed of ten microbial species: eight bacteria and two yeasts (Table 1). The organisms in CS (hereafter referred to as 'Even') are distributed equally (12%), with the exception of the two yeasts which are each present at 2%. Cell counts from organisms in CSII ('Log') community are distributed on a log scale, ranging from 89.1% (*Listeria monocytogenes*), down to 0.000089% (*Staphylococcus aureus*).

Methods

DNA extraction

DNA was extracted from 75 µl ZymoBIOMICS Microbial Community Standard (Product D6300, Lot ZRC190633) and 375 µl ZymoBIOMICS Microbial Community Standard II (Product D6310, Lot ZRC190842) using the ZymoBIOMICS DNA Miniprep extraction kit according to manufacturer's instructions, with the following modifications to increase fragment length and maintain the expected representation of the Gram-negative species which are already lysed in the DNA/RNA Shield storage solution. The standard was spun at 8,000×*g* for 5 minutes before removing the supernatant and retaining. The cell pellet was resuspended in 750 µl lysis buffer and added to the ZR BashingBead lysis tube. Bead-beating was performed on a FastPrep-24 (MP Biomedicals) instrument for 2 cycles of 40 seconds at power level 6, with 5 minutes sitting on ice between cycles. The bead tubes were spun at 10,000×*g* for 1 minute and 450 µl of supernatant was transferred to a Zymo Spin III-F filter before being spun again at 8000×*g* for 1 minute. 45 µl (Even) and 225 µl (Log) of the supernatant retained earlier was combined with 450 µl filtrate before adding 1485 µl (Even) or 2025 µl (Log) Binding Buffer and mixing before loading onto the column.

Nanopore sequencing library preparation

Quantification steps were performed using the dsDNA HS assay for Qubit. DNA was size-selected by cleaning up with 0.45× volume of Ampure XP (Beckman Coulter) and eluted in 100 µl EB (Qiagen). Libraries were prepared from 1400 ng input DNA using the LSK-109 kit (Oxford Nanopore Technologies) as

Table 2. Summary of the four nanopore sequencing experiments.

Signal Accession	FASTQ Accession	Sequencer	Standard	Time (h)	Reads (M)	N50 (Kbp)	Quality (Median Q)	Yield (Gbp)	Q>7 (Gbp)
ERR2887847	ERR2906227	GridION	Zymo CS Even ZRC190633	48	3.49	5.3	9.8	14.01	12.31
ERR2887850	ERR2906229	GridION	Zymo CSII Log ZRC190842	48	5.73	5.2	9.3	16.03	13.67
ERR2887848<br>ERR2887849	ERR2906228	PromethION<br>PromethION	Zymo CS Even ZRC190633<br>Zymo CS Even ZRC190633	64<br>–	36.5	5.3	9.1	146.29	123.27
ERR2887851<br>ERR2887852	ERR2906230	PromethION<br>PromethION	Zymo CSII Log ZRC190842<br>Zymo CSII Log ZRC190842	64<br>–	35.1	5.2	9.2	148.03	126.12

PromethION runs were restarted following the standard 64 hour protocol. The table reflects total yield across both the standard run and subsequent restarts.

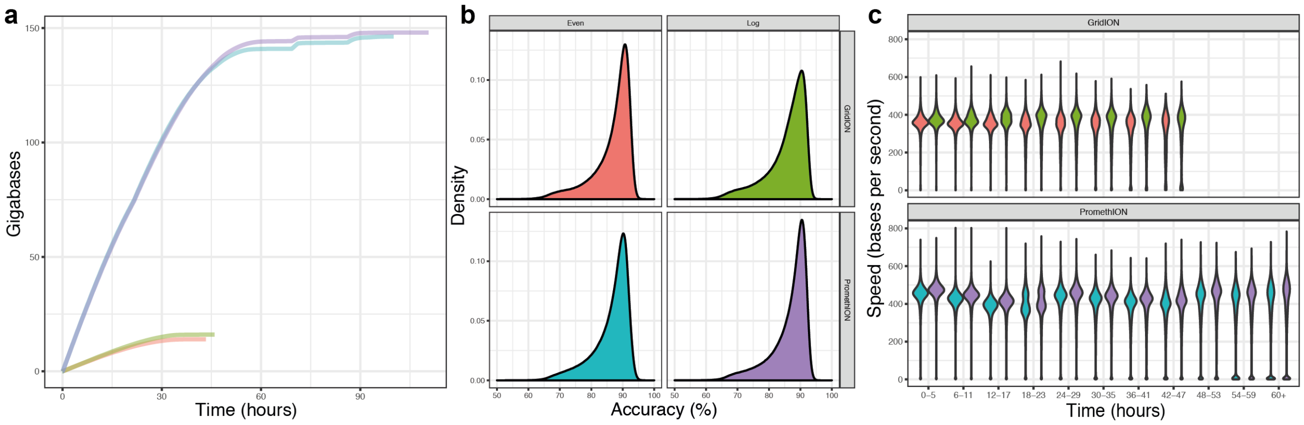

Figure 1. Summary plots for the four generated data sets: (a) collector's curve showing sequencing yield over time for each of the four sequencing runs, (b) density plot showing sequence accuracy (BLAST-like identities), (c) violin plot showing sequencing speed over time split by sequencing group.

per manufacturer's protocol, except incubation times for end-repair, dA-tailing and ligation were increased to 30 minutes to improve ligation efficiency. The even and log libraries were split and used on both the GridION and PromethION flowcells.

Sequencing

Sequencing libraries were quantified and two aliquots of 50 ng and 400 ng were prepared for GridION and PromethION sequencing respectively. The GridION sequencing was performed using a `FLO-MIN106` (rev.C) flowcell, `MinKNOW` 1.15.1 and standard 48-hour run script with active channel selection enabled. The PromethION sequencing was performed using a `FLO-PRO002` flowcell, `MinKNOW` 1.14.2 and standard 64-hour run script with active channel selection enabled.

Refuelling was performed approximately every 24h (GridION, PromethION) by loading 75 µl (GridION) or 150 µl (PromethION) refuelling mix (SQB diluted 1:2 with nuclease-free water). Additionally, after the standard scripts had completed the PromethION was restarted several times to utilise remaining active pores and maximise total yield.

Nanopore basecalling

Reads were basecalled on-instrument using the GPU basecaller `Guppy` (Oxford Nanopore Technologies) with the supplied `dna_r9.4.1_450bps_prom.cfg` configuration (PromethION) and `dna_r9.4_450bps.cfg` (GridION). `Guppy` version 1.8.3 and 1.8.5 were installed on the GridION and PromethION respectively.

Illumina sequencing

DNA was extracted from pure cultures of each species using the ZymoBIOMICS DNA Miniprep Kit. Library preparation was performed using the Kapa HyperPlus Kit with 100 ng DNA as input and TruSeq Y-adapters. The purified library derived from each sample was quantified by TapeStation (Agilent 4200) and pooled together in an equimolar fashion. The pooled library was sequenced on an Illumina HiSeq 1500 instrument using 2×101 bp (paired-end) sequencing, over four lanes. Raw reads were demultiplexed using `bcl2fastq` v2.17. Shotgun sequencing of the even and log communities was performed with the same protocol, with the exception that the log community was sequenced individually on two flowcell lanes, and the even community was instead sequenced on an Illumina MiSeq using 2×151 bp (paired-end) sequencing.

Bioinformatics

To construct reference genomes, Illumina reads for each of the ten isolates were assembled using `SPAdes` v3.12.0 [16] with paired-end reads as input, using parameters `-m 512 -t 12`. Scaffolds from `SPAdes` less than 500 bp length or with less than 10× coverage were removed. The remaining scaffolds were combined into a single mock community draft assembly for downstream analysis. Multilocus sequence typing (MLST) of the scaffolds was conducted with `mlst` (https://github.com/tseemann/mlst), using default parameters.

Nanopore reads were aligned to the mock community draft assembly using `minimap2` [17] v2.14-r883 with parameters `-ax map-ont -t 12` and converted to a sorted BAM file using `samtools` [18]. To reduce erroneous mappings, alignment BAM files were filtered using a script `bamstats.py` according to the following criteria; reference mapping length ≥500 bp, map quality (MAPQ) > 0, there are no supplementary alignments for this read and read is not a secondary alignment. Per-species coverage summary statistics were generated using the `summariseStats.R` Rscript.

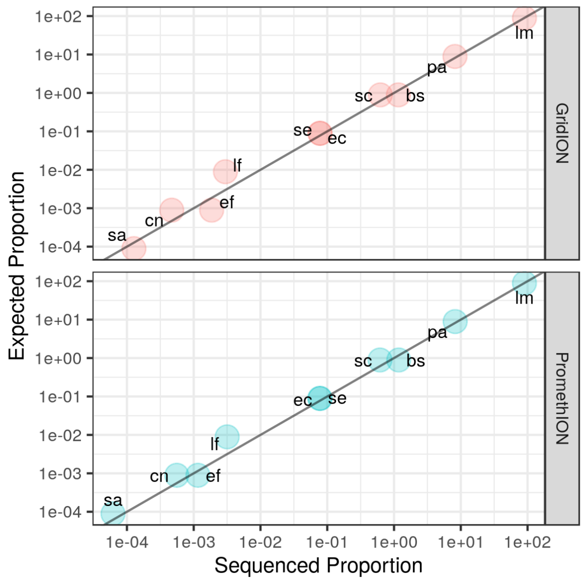

Figure 2. Proportion of sequenced DNA from each of the 10 organisms that was sequenced (x-axis), against the proportion of yield expected given the known community composition (y-axis) of the Zymo CSII (Log) standard.

Table 3. Read alignment statistics for even samples, showing absolute measurements and proportion of sequencing yield and the estimated genome coverage obtained for each organism in the mock community.

Species	Expected Proportion	GridION Yield (Gbp)	GridION Measured Proportion	GridION Aln. N50 (Kbp)	GridION Coverage (×)	PromethION Yield (Gbp)	PromethION Measured Proportion	PromethION Aln. N50 (Kbp)	PromethION Coverage (×)
Bacillus subtilis	12	2.09	19.30	4.30	516.41	21.54	18.98	4.40	5325.31
Listeria monocytogenes	12	1.57	14.53	4.46	525.44	16.20	14.28	4.57	5414.09
Enterococcus faecalis	12	1.32	12.21	4.45	464.33	13.66	12.04	4.56	4802.85
Staphylococcus aureus	12	1.22	11.25	4.46	445.98	12.58	11.08	4.57	4607.07
Salmonella enterica	12	1.08	10.01	8.56	227.50	11.76	10.36	8.94	2470.85
Escherichia coli	12	1.08	9.95	8.31	220.93	11.69	10.30	8.70	2397.58
Pseudomonas aeruginosa	12	1.05	9.73	9.00	155.08	11.58	10.20	9.37	1704.32
Lactobacillus fermentum	12	1.01	9.30	3.6	528.46	10.35	9.12	3.72	5433.40
Saccharomyces cerevisiae	2	0.21	1.92	4.08	17.18	2.12	1.87	4.17	174.97
Cryptococcus neoformans	2	0.19	1.79	4.44	10.23	2.00	1.76	4.53	105.95

Table 4. Read alignment statistics for log samples, describing sequencing yield and estimated genome coverage obtained for each organism in the mock community.

Species	GridION Yield (Gbp)	GridION Aln. N50 (Kbp)	GridION Coverage (×)	PromethION Yield (Gbp)	PromethION Aln. N50 (Kbp)	PromethION Coverage (×)
Listeria monocytogenes	11.85	4.94	3960.85	109.69	4.97	36 659.72
Pseudomonas aeruginosa	1.08	9.38	158.83	10.06	9.33	1481.29
Bacillus subtilis	0.15	5.02	37.89	1.44	5.03	355.39
Saccharomyces cerevisiae	0.08	4.76	6.79	0.75	4.74	62.19
Salmonella enterica	0.01	9.26	2.15	0.10	9.14	20.09
Escherichia coli	0.01	8.72	2.11	0.09	9.17	19.33
Lactobacillus fermentum	4×10^{-4}	3.39	0.207	0.004	3.35	2.03
Enterococcus faecalis	2×10^{-4}	7.50	0.086	1×10^{-4}	6.81	0.50
Cryptococcus neoformans	6×10^{-5}	4.39	0.003	7×10^{-4}	4.96	0.036
Staphylococcus aureus	2×10^{-5}	3.87	0.006	8×10^{-5}	3.03	0.028

Note that expected and measured proportions are illustrated by Figure 2.

Read accuracy was determined by calculating BLAST identity from the filtered alignments (as per http://lh3.github.io/2018/11/25/on-the-definition-of-sequence-identity), calculated as $(L - NM)/L$ using the minimap2 number of mismatches (NM) SAM tag and the sum of match, insertion and deletion CIGAR operations (L).

We used wtdbg2 v2.2 to assemble nanopore data (https://github.com/ruanjue/wtdbg2). wtdbg2 was compiled from source in November 2018 from Git commit 094f2b3. For GridION, all nanopore reads were used. For PromethION, we used both the full dataset and a random 25% subsample. Subsampling was performed using subsample.py.

Assemblies were conducted under a variety of parameter values for homopolymer-compressed k-mer size (-p), minimum graph edge weight support (-e) and read length threshold (-L). Global parameters for all runs (-S1 -K10000 -node-max 6000) were used to turn-off k-mer subsampling (to remove assembly stochasticity) and increase the coverage thresholds applied to k-mers and constructed nodes.

Table 5. Summary statistics for Illumina sequencing data.

Dataset	Pairs (M)	Yield (Gbp)	phred≥30 (%)	Accession
Isolates	13.53 ± 5.23	2.73 ± 1.06	87.72% ± 5.43%	see Table 1
CS (Even)	8.8	2.65	95.12 %	ERR2984773
CSII (Log)	47.8	9.66	95.71 %	ERR2935805

Illumina sequencing was performed on an Illumina HiSeq 1500, with the exception of the even community which was sequenced on an Illumina MiSeq.

Assembled contigs were assigned to taxa with kraken2 [19] (--use-names -t12) using a database containing all of the archaeal, bacterial, fungal, protozoal and viral sequences from RefSeq, and UniVec_Core (database download links are in our repository). The kraken2 output was parsed with extracken.py. Assembly accuracy was determined by a modified version of fastmer.py (https://github.com/jts/assembly_accuracy) which uses minimap2 to align contigs against the draft assembly.

Results

Nanopore sequencing metrics

We generated a total of 324.36 Gbp of sequence from the four nanopore sequencing runs (Table 2, Figure 3.a). PromethION flowcells generated approximately ten-times more sequencing data than the comparative GridION runs and showed equivalent read length N50 and quality scores (Figure 1.b). We observe a difference in sequencing speed between the PromethION (mean 395 bps and 412 bps for even and log) and the GridION (mean speed 341 and 359 bps for even and log) (Figure 1.c).

Illumina sequencing metrics

Illumina datasets for the ten individually sequenced isolates averaged 13.53 million pairs of reads (ranging between 7.1 – 23.2 million), with proportions of reads with a mean phred score ≥30 ranging between 75.51% – 93.09% (Table 5). Illumina sequencing generated 8.8 million pairs of reads (2×151 bp, MiSeq), and 47.8 million pairs of reads (2×101 bp, HiSeq) for the even and log community, respectively (Table 5).

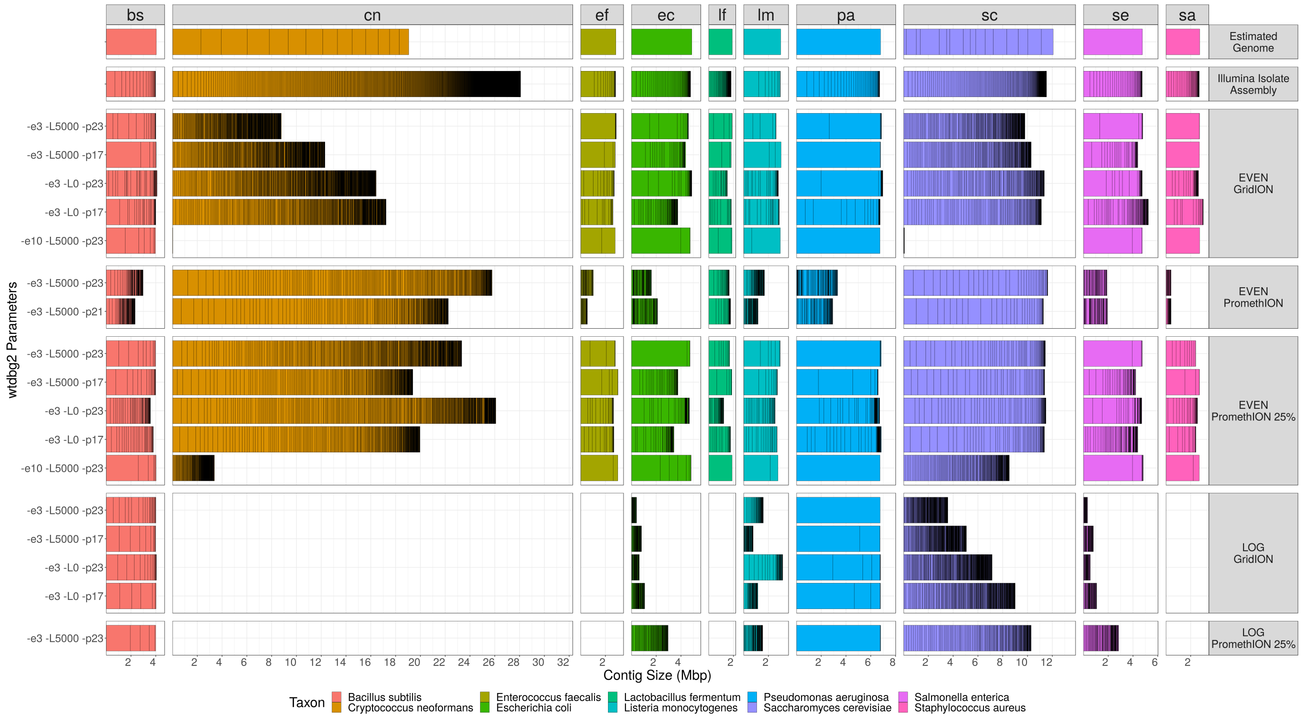

Figure 3. Bar plots demonstrating total length and contiguity of genomic assemblies obtained with `wtdbg2` from each of the long-read nanopore data sets. For each organism in the community (coloured columns), contigs longer than 10 kbp are horizontally stacked along the x-axis. Each row represents a run of `wtdbg2`, with the parameters for edge support, read length threshold and homopolymer-compressed k-mer size labelled on the left. Assemblies are grouped by the data set on which they were run (row facets). Additionally, assemblies may be compared to the estimated true genome size, and per-isolate Illumina SPADES assembly. Estimated genomes sizes are the same as those found in Table 1, however to display approximate chromosomes, the two yeasts were replaced by their corresponding canonical NCBI references for visualisation purposes only. The *C. neoformans* strain used by the Zymo standards is a diploid genetic cross, which may explain the larger assemblies, compared to the represented estimated haploid size.

Nanopore mapping statistics

We identify the presence of all 10 microbial species in the community, for both even and log samples, in expected proportions (Figure 2). For the even community, the GridION results provide sufficient depth (i.e. $\gg 30\times$ coverage) to potentially assemble all eight of the bacteria. The coverage of the yeast genomes were lower (10 and 17×), potentially sufficient for assembly scaffolding. On the PromethION all genomes had >100× mean coverage (Tables 3 and 4).

For the log-distributed community, three taxa have sufficient coverage for assembly on GridION, compared with four on PromethION. On PromethION, a further two genomes (*S. enterica* and *E. coli*) have sufficient coverage for assembly scaffolding. We are able to detect *S. aureus*, the lowest abundance organism on both platforms, with 32 reads from PromethION (from 450 cell input) and 7 reads from GridION (from 50 cell input).

Nanopore metagenome assemblies

We evaluated nanopore metagenomic assemblies and assessed genome completeness and contiguity for each run with different assembly parameters.

For the even community, genomes of the expected size were present for each of the bacterial species contained in small numbers of large contigs (Figure 3). However, the two yeasts are poorly represented in the assembly, consistent with their low read depth.

L. monocytogenes is poorly assembled in the log dataset despite being the most abundant organism, indicating very high sequence coverage may be detrimental to the performance of `wtdbg2`. We also observe that assembling the entire PromethION dataset resulted in less complete and more fragmented assemblies. This led us to sample randomly the PromethION data to 25% of the total dataset which improved the assembly results.

After subsampling, assemblies of the even community from the GridION and PromethION are similar. However, the assemblies from PromethION data had better representation of the yeasts (particularly *C. neoformans*), due to the higher coverage depths of these species.

Average consensus accuracy was calculated for the entire set of assemblies (n=51). For the even community, accuracy is 99.1% (± 0.17%) and 99.0% (± 0.24%) for the GridION and PromethION respectively. For the log community, average accuracy is 99.0% (± 0.27%) and 99.1% (± 0.20%) for the GridION and PromethION respectively.

Discussion

There are several noteworthy aspects of this dataset: We generated nearly 300 Gbp of sequence data from the Oxford Nanopore PromethION and 30 gigabases from the Oxford Nanopore GridION, on a well-characterised mock community sample and we have made basecalls and electrical signal data for each of the four runs presented here available: a combined dataset size of over four terabytes. The availability of the raw signal permits future basecalling of the data (an area under rapid development), as well as signal-level polishing and the detection of methylated bases [20].

Individual sequencing libraries were split between the GridION and PromethION, permitting direct comparisons of the instruments to be made. We observed high concordance between the datasets from each platform. We note the sequencing speed of the PromethION is faster than the GridION, which we attribute to different running temperatures on these instrument (39°C versus 34°C, respectively).

Confident detection of *S. aureus* was demonstrated for the GridION run to <50-cells using the log community. The PromethION generated around five times the number of *S. aureus* reads as the GridION, but appears less sensitive as we loaded eight times more library. However, it may be possible to reduce the loading input to PromethION flowcells, but we have not attempted this.

Early results of metagenomics assembly show promise for reconstruction of whole microbial genomes from mixed samples without a binning step. We focused on the recently released `wtdbg2` software as we found that the established `minimap2` and `miniasm` method resulted in excessively large intermediate files (tens of terabases per analysis) which were impractical to store and analyse.

For the even community, using `wtdgb2` with varying parameter choices, we were able to assemble seven of the bacteria into single contigs. However, no single parameter set was found to be optimum for both total genome size and contig length.

We found that `wtdbg2` expects a maximum of 200× sample coverage, and discards sequence k-mers and *de Bruijn* graph nodes with more than 200× support. These limits must be lifted by specifying higher `-K` and `-node-max` for this dataset. Increasing `-e` improved contiguity for the even sample, however this resulted in the loss of yeasts from the assembly. Increasing the read length threshold (`-L`) improved assembly contiguity for all sample and platform combinations, at the cost of genome size. Increasing the homopolymer-compressed k-mer size (`-p`) from the default of 21 to 23 also appears to improve assembly contiguity. It should be noted that `wtdbg2` is still under active development, making it difficult to make concrete recommendations for parameters.

The availability of this dataset should help with further improvements to long-read assembly techniques.

This study has several shortcomings:

We have not yet explored polishing techniques to improve consensus accuracy of assemblies using nanopore or Illumina data [21]. A new "flip-flop" basecaller, `Flappie` (https://github.com/nanoporetech/flappie), was recently made available although we have not used it on this dataset.

Although reference genomes for the ten microbial strains contained in the standards are available from Zymo (https://s3.amazonaws.com/zymo-files/BioPool/ZymoBIOMICS.STD.refseq.v2.zip), they are constructed from a combination of nanopore and Illumina data (not presented here). To avoid a circular comparison, we chose to perform analysis only against Illumina draft genomes.

Other mock microbial samples are available which we did not test here. A notable alternative mock community sample is from the Human Microbiome Project (HMP) and consists of 20 microbial samples (available from BEI Resources). This mock community have been sequenced as part of other studies, although the datasets are much smaller than the ones presented here [9, 22]. Bertrand et al. presented a synthetic mock community of their own construction to demonstrate hybrid nanopore-Illumina metagenome assemblies [12].

Re-use potential

The provision of Illumina reads for each isolate permits a ground-truth to be obtained for the individual species contained in the mock community. This will be useful for training new nanopore basecalling and polishing models, long-read aligners, variant callers, and validating taxonomic assignment and assembly software and pipelines.

Availability of source code and requirements

Python and R scripts used to generate the summary information and analyses are open source and freely available via our repository (https://github.com/LomanLab/mockcommunity), under the MIT license.

Availability of supporting data and materials

This manuscript, and its supporting data are available under a Creative Commons Attribution 4.0 International license.

Unprocessed FASTQ from the Illumina sequencing of the ten isolates are available at the European Nucleotide Archive, via the identifiers listed in Table 1, identifiers for the even and log community Illumina sequencing can be found in Table 5.

Both the raw signal, and basecalled FASTQ for the nanopore sequencing experiments are available at the European Nucleotide Archive, via the identifiers listed in Table 2.

The `SPAdes`-assembled Illumina draft reference, and the collection of nanopore assemblies for each `wtdbg2` condition are linked to from our GitHub repository (https://github.com/LomanLab/mockcommunity), along with the `kraken2` database used for taxonomic classification of the assembled contigs.

Further updates (such as updated references, or new assemblies) will be made available through our project website https://lomanlab.github.io/mockcommunity/.

Declarations

Consent for publication

Not applicable

Competing Interests

Cambridge Biosciences provided the ZymoBIOMICS Microbial Community Standard free of charge. ST is an employee of Zymo Research Corporation. NJ has received Oxford Nanopore Technologies (ONT) reagents free of charge to support his research programme. NJ and JQ have received travel expenses to speak at ONT events. NL has received an honorarium to speak at an ONT company meeting.

Funding

SN is funded by the Medical Research Foundation and the NIHR STOP-COLITIS project. JQ is funded by the NIHR Surgical Reconstruction and Microbiology Research Centre. The NIHR SRMRC is a partnership between The National Institute for Health Research, University Hospitals Birmingham NHS Foundation Trust, the University of Birmingham, and the Royal Centre for Defence Medicine. NL is funded by an MRC Fellowship in Microbial Bioinformatics under the CLIMB project.

Author's Contributions

Conceptualization: NL, Methodology: NL JQ SN ST, Software: SN NL, Validation: SN NL, Formal analysis: SN NL, Investigation: NL JQ SN, Resources: NL ST, Data Curation: SN NL ST, Writing – original draft preparation: SN, Writing – review and editing: SN NL JQ ST, Visualization: SN NL, Supervision: NL, Project administration: NL, Funding acquisition: NL ST

Acknowledgements

We are grateful to Radoslaw Poplawski (University of Birmingham) for assistance with CLIMB virtual machines and file systems to support this research. We thank Divya Mirrington (Oxford Nanopore Technologies) for advice on PromethION library preparation and sequencing. We thank Hannah McDonnell at Cambridge Biosciences for providing the ZymoBIOMICS Microbial Community Standards. We thank Jared Simpson (Ontario Institute for Cancer Research), Matt Loose (University of Nottingham) and John Tyson (University of British Columbia) for useful discussions and advice.

References

1. Handelsman J. Metagenomics: application of genomics to uncultured microorganisms. Microbiology and Molecular Biology Reviews 2004 Dec;68(4):669–685.
2. Hug LA, Baker BJ, Anantharaman K, Brown CT, Probst AJ, Castelle CJ, et al. A new view of the tree of life. Nature Microbiology 2016 Apr;1:16048.
3. Quince C, Walker AW, Simpson JT, Loman NJ, Segata N. Shotgun metagenomics, from sampling to analysis. Nature Biotechnology 2017 Sep;35(9):833–844.
4. Jain M, Koren S, Miga KH, Quick J, Rand AC, Sasani TA, et al. Nanopore sequencing and assembly of a human genome with ultra-long reads. Nature Biotechnology 2018 Jan;36:338.
5. Payne A, Holmes N, Rakyan V, Loose M. BulkVis: a graphical viewer for Oxford nanopore bulk FAST5 files. Bioinformatics 2018;p. bty841.
6. Sanderson ND, Street TL, Foster D, Swann J, Atkins BL, Brent AJ, et al. Real-time analysis of nanopore-based metagenomic sequencing from infected orthopaedic devices. BMC Genomics 2018 Sep;19(1):714.
7. Charalampous T, Richardson H, Kay GL, Baldan R, Jeanes C, Rae D, et al. Rapid Diagnosis of Lower Respiratory Infection using Nanopore-based Clinical Metagenomics. bioRxiv 2018;p. 387548.
8. Somerville V, Lutz S, Schmid M, Frei D, Moser A, Irmler S, et al. Long read-based de novo assembly of low complex metagenome samples results in finished genomes and reveals insights into strain diversity and an active phage system. bioRxiv 2018;p. 476747.
9. Leggett RM, Alcon-Giner C, Heavens D, Caim S, Brook TC, Kujawska M, et al. Rapid profiling of the preterm infant gut microbiota using nanopore sequencing aids pathogen diagnostics. bioRxiv 2018;p. 180406.
10. Huson DH, Auch AF, Qi J, Schuster SC. MEGAN analysis of metagenomic data. Genome Research 2007 Mar;17(3):377–386.
11. Wommack KE, Bhavsar J, Ravel J. Metagenomics: read length matters. Applied and Environmental Microbiology 2008 Mar;74(5):1453–1463.
12. Bertrand D, Shaw J, Narayan M, Ng HQA, Kumar S, Li C, et al. Nanopore sequencing enables high-resolution analysis of resistance determinants and mobile elements in the human gut microbiome. bioRxiv 2018;p. 456905.
13. Sczyrba A, Hofmann P, Belmann P, Koslicki D, Janssen S, Dröge J, et al. Critical assessment of metagenome interpretation—a benchmark of metagenomics software. Nature Methods 2017;14(11):1063.
14. Mason CE, Afshinnekoo E, Tighe S, Wu S, Levy S. International Standards for Genomes, Transcriptomes, and Metagenomes. Journal of Biomolecular Techniques 2017 Apr;28(1):8–18.
15. Ackelsberg J, Rakeman J, Hughes S, Petersen J, Mead P, Schriefer M, et al. Lack of Evidence for Plague or Anthrax on the New York City Subway. Cell Systems 2015 Jul;1(1):4–5.
16. Bankevich A, Nurk S, Antipov D, Gurevich AA, Dvorkin M, Kulikov AS, et al. SPAdes: a new genome assembly algorithm and its applications to single-cell sequencing. Journal of Computational Biology 2012 May;19(5):455–477.
17. Li H. Minimap2: pairwise alignment for nucleotide sequences. Bioinformatics 2018;1:7.
18. Li H, Handsaker B, Wysoker A, Fennell T, Ruan J, Homer N, et al. The sequence alignment/map format and SAMtools. Bioinformatics 2009;25(16):2078–2079.
19. Wood DE, Salzberg SL. Kraken: ultrafast metagenomic sequence classification using exact alignments. Genome Biology 2014 Mar;15(3):R46.
20. Simpson JT, Workman RE, Zuzarte PC, David M, Dursi LJ, Timp W. Detecting DNA cytosine methylation using nanopore sequencing. Nature Methods 2017 Apr;14(4):407–410.
21. Rang FJ, Kloosterman WP, de Ridder J. From squiggle to basepair: computational approaches for improving nanopore sequencing read accuracy. Genome Biology 2018 Jul;19(1):90.
22. Huson DH, Albrecht B, Bağcı C, Bessarab I, Górska A, Jolic D, et al. MEGAN-LR: new algorithms allow accurate binning and easy interactive exploration of metagenomic long reads and contigs. Biology Direct 2018 Apr;13(1):6.
