# Supplementary material for: Ultra-deep, long-read nanopore sequencing of mock microbial community standards
Source: Gigascience. 2019 May 15;8(5):giz043. doi: 10.1093/gigascience/giz043 (PMC6520541; doi:10.1093/gigascience/giz043)
Supplement: GIGA-D-18-00495_Revision_1.pdf [file giz043_giga-d-18-00495_revision_1.pdf]

GigaScience

# Ultra-deep, long-read nanopore sequencing of mock microbial community standards

--Manuscript Draft--

| | |
|---|---|
| Manuscript Number: | GIGA-D-18-00495R1 |
| Full Title: | Ultra-deep, long-read nanopore sequencing of mock microbial community standards |
| Article Type: | Data Note |
| Funding Information: | |
| Abstract: | Long sequencing reads are information-rich: aiding de novo assembly and reference mapping, and consequently have great potential for the study of microbial communities. However, the best approaches for analysis of long-read metagenomic data are unknown. Additionally, rigorous evaluation of bioinformatics tools is hindered by a lack of long-read data from validated samples with known composition.<br><br>Methods: We sequenced two commercially-available mock communities containing ten microbial species (ZymoBIOMICS Microbial Community Standards) with Oxford Nanopore GridION and PromethION. Both communities and the ten individual species isolates were also sequenced with Illumina technology.<br><br>Data: We generated 14 and 16 Gbp from two GridION flowcells and 150 and 153 Gbp from two PromethION flowcells for the evenly-distributed and log-distributed communities respectively. Read length N50 ranged between 5.3 Kbp and 5.4 Kbp over the four sequencing runs. Basecalls and corresponding signal data are made available (4.2 TB in total).<br><br>Results: Alignment to Illumina-sequenced isolates demonstrated the expected microbial species at anticipated abundances, with the limit of detection for the lowest abundance species below 50 cells (GridION). De novo assembly of metagenomes recovered long contiguous sequences without the need for pre-processing techniques such as binning.<br><br>Conclusions: We present ultra-deep, long-read nanopore datasets from a well-defined mock community. These datasets will be useful for those developing bioinformatics methods for long-read metagenomics and for the validation and comparison of current laboratory and software pipelines. |
| Corresponding Author: | Nicholas James Loman<br><br>UNITED KINGDOM |
| Corresponding Author Secondary Information: | |
| Corresponding Author's Institution: | |
| Corresponding Author's Secondary Institution: | |
| First Author: | Samuel M Nicholls |
| First Author Secondary Information: | |
| Order of Authors: | Samuel M Nicholls |
| | Joshua C Quick |
| | Shuiquan Tang |
| | Nick Loman |
| Order of Authors Secondary Information: | |
| Response to Reviewers: | Dear Editor,<br>Since our previous submission, our manuscript has been updated. In particular we have re-performed basecalling on the original nanopore data with an updated |

Powered by Editorial Manager® and ProduXion Manager® from Aries Systems Corporation

basecaller. Additionally, we have made comparisons between our long-read assemblies and some recently released PacBio assemblies of individuals from the same mock community. We believe this addresses the primary concern of our reviewers.

We provide detailed responses to the specific comments of our reviewers below.

**

Reviewer 1

R1.1. It seems that species assignment was done in two ways, one by using Kraken on the contigs (with a database of many bacterial/viral/fungal genomes) ; and also by mapping the reads directly to the illumina assemblies of the isolates in the mixture. It would be useful to be clearer in the results which approach was used in reporting the results.

E.g. the sentence "We identify the presence of all 10 microbial species in the community, for both even and log samples, in expected proportions (Figure 2)." presumably relates to the analysis just mapping to the draft illumina assemblies?

Response 1.1: We agree that the manuscript could be clearer in this regard. The read-level analysis identified taxa by mapping to a combined Illumina assembly of all the isolates. The assembly-level analysis used kraken2 to assign contigs to species. We have clarified our Methods; in particular, the “Bioinformatics” subsection of the Methods has been elevated to a section of its own, allowing us to add suitable subheadings to better delimit our computational methods.

R1.2. Also, It seems a little surprising that there were no false positive identification of species not present in the mixture. Is this because this analysis is based on mapping to the draft illumina isolate assemblies only (see previous comment). Or, if based on kraken assignment of contigs, perhaps repetitive and/or short contigs were filtered out?

Response 1.2: The reviewer is correct that this is because analysis is based on mapping to the Illumina assemblies only with minimap2. If kraken2 had been used on individual reads, some level of misassignment would be expected.

R1.3. Could the authors present more statistics on the quality of the nanopore metagenomic assemblies, including the presence of misassemblies, any chimeric contigs, checkM completeness results; indel errors, mismatch errors, etc.

Response 1.3: We thank the reviewer for this helpful suggestion. In our initial manuscript this was difficult because of the absence of high quality whole-genome assemblies that would permit orthogonal assessment of assembly correctness. However, in the past weeks, PacBio assemblies of 7 out of 8 bacteria present in the Zymo community (excluding Lactobacillus fermentum) have been made available in McIntyre et al. Nature Communications 2019. We were able to use these genomes to assess correctness of our assemblies. Although PacBio draft assemblies are available also for S. cerevisiae (but not C. neoformans), we did not include it in the comparison because they were still heavily fragmented.

We have updated the manuscript with extensive new results (Table 6). This table demonstrates dotplots of our assembled metagenomic contigs to these new PacBio references, permitting assessment of misassemblies. Additionally, Table 6 demonstrates the CheckM completeness score for the 7 bacteria that have a corresponding PacBio assembly. Because the CheckM score is heavily contingent on consensus-level sequence identity and reflects presence of indel errors, we have included completeness results for common polishing pipelines: Racon (x2), plus Medaka, plus Illumina polishing with Pilon (x2).

R1.4. Also, can the authors confirm that the assemblies were done on the full nanopore dataset (rather than, for example, on each isolate separately after mapping the reads to each isolate draft illumina assembly).

Powered by Editorial Manager® and ProduXion Manager® from Aries Systems Corporation

| | |
|---|---|
| | Response 1.4: We can confirm that the assemblies are constructed from nanopore sequencing of the entire community (using 100% of GridION data, and a random 25% subsampling of PromethION sequence data). No read binning or pre-assignment steps were employed. We hope that our changes described in Response 1.1 have clarified the manuscript for readers.<br><br><br>R1.5. The authors write: "For the even community, using wtdgb2 with varying parameter<br>choices, we were able to assemble seven of the bacteria into single contigs." , however this does not seem to be borne out by figure 3? I could only see 4 species with at least one single contig assembly. Perhaps the authors could spell out which species have a single contig assembly?<br><br>Response 1.5: Our assessments were initially conducted by inspecting the contiguity visualisation (Figure 3). However, as we now have access to an orthogonal source of reference genomes (as described in Response 1.3), we have been able to construct sequence identity dotplots, and calculate the L50 and L95 for our assemblies. We believe for the GridION data there are 4 species for which we yield a single contig assembly (Table 6) and have updated our manuscript with this figure. The contiguity plot suggests that for at least one assembly condition, we recover L. fermentum in a single contig, but we do not have the corresponding PacBio reference. We thank the reviewer for suggesting this statement be clarified with more evidence, we believe Table 6 addresses the issues raised.<br><br><br>R1.6. In abstract "even and odd communities" should be ' evenly-distributed and log-distributed communities for clarity (this term is otherwise unclear to casual reader of abstract)<br><br>Response 1.6: The incorrect reference to the “odd community” has been corrected.<br><br>**<br><br>Reviewer 2<br>R2.1. Line 28-29: Would suggest that the authors provide the citation (15) without the statement in parenthesis or revised version of statement in parenthesis.<br><br>Response 2.1: We thank the reviewer for this comment, the manuscript has been revised accordingly.<br><br><br>R2.2. "DNA extraction protocol" section: The last few lines were a little bit unclear. For instance: "45 ul (Even) and 225ul (Log) of the supernatant retained earlier…" It was a bit confusing. Possibly because the line "The standard was spun…before removing the supernatant and retaining." seems incomplete. I would suggest that the authors consider posting the entire protocol on protocols.io - as is quite possible that other groups may want to reproduce the sequencing step for these mock community standards. This would be particularly helpful as the authors suggest that the protocol was modified to increase fragment length.<br><br>Response 2.2: We agree the manuscript could be clearer in this section and have clarified the points raised.<br>We thank the reviewer for suggesting we post the DNA extraction methods on protocols.io, we have made the method available via dx.doi.org/10.17504/protocols.io.x9tfr6n.<br><br><br>R2.3. "Illumina sequencing" section: Suggest that the authors improve clarity in this section by re-structuring this paragraph. For instance, early in paragraph it is stated that the pooled library was sequenced on four lanes on Illumina HiSeq 1500, but later stated that the even community was sequenced on a MiSeq.<br><br>Response 2.3: The Illumina sequencing section has been edited for clarity as |

Powered by Editorial Manager® and ProduXion Manager® from Aries Systems Corporation

suggested. The phrase “pooled library” has been changed to “multiplexed isolates” to distinguish the simultaneous sequencing of the individual isolates as different from the shotgun sequencing of the even community itself.

R2.4. “Table 2, Figure 3a”. - please fix this to Figure 1a.

Response 2.4: Thank you for noticing this incorrect reference, it has been fixed.

R2.5. Figure 1B: The x-axis is "accuracy" while in this section Figure 1b is referred to as providing "quality scores". Please replace "quality scores" with "accuracy" for consistency.

Response 2.5: The paragraph has been updated for consistency.

R2.6. Figure 1C: Please provide a legend mapping colors to "even" and "log". I realize this information is in Figure 1B, but would be helpful for the reader. Finally, there is no significant trend in sequencing speed over time. Considering this, would be easier to remove the Time component and just have a single panel with the GridION and PromethION sequencing speed for both even and log community in the same panel. It would make it easier to compare the different in sequencing speeds visually.

Response 2.6: We thank the reviewer for this useful comment. Figure 1c has been replaced with a simpler density plot showing the distribution of sequencing speeds for the four runs, and we have also added a suitable colour-coded legend for the reader.

R2.7. Table 5 is mentioned before Tables 3 and 4. Please correct this.

Response 2.7: Table 5 has been moved to earlier in the manuscript and is now referred to as Table 3.

R2.8. For Figure 2, consider also providing figure for the even community.

Response 2.8: We appreciate this comment but feel that the information for the even community is better represented by the numerical values provided in Table 4.

R2.9. Further, it would be helpful to get clarity on where the data for Figure 2 is coming from. Is this from mapping of long-reads to mock community draft (I think so) or from the kraken analyses.

Response 2.9: Our reviewer is correct that the data was from the mapping of long-reads to the Illumina mock community draft, and we agree that the caption could be clearer. The figure legend has been clarified to explicitly state the mapping method used.

R2.10. It is unclear how the genome completeness was estimated.

Response 2.10: The latest version of the manuscript now formally estimates genome completeness using measurements of L50/L95 against the reference genome, and CheckM completeness estimates (as described more fully in Response 1.3). The Methods section has been updated accordingly.

R2.11. The consensus accuracy data is provided for all assemblies combined. Would be helpful if there was some discussion on accuracy of assemblies as a function of wtdgb2 parameters tested. There is some discussion of this in the "Discussion section", but would be helpful if this was laid out clearly in the results, with an additional appropriate figure/table.

Powered by Editorial Manager® and ProduXion Manager® from Aries Systems Corporation

| | |
|---|---|
| | Response 2.11: We agree that the manuscript would benefit from a presentation of assembly accuracy. As described in Response 1.3, with the availability of a recently released orthogonal data set, we can now estimate accuracy and genome completeness. We believe Table 6 lays out these results clearly.<br><br>**<br><br>We thank the reviewers for their insightful and constructive feedback, which has helped us improve the manuscript, and for this opportunity to respond.<br><br><br>Sam Nicholls & Nicholas Loman<br>(On behalf of the Authors) |
| **Additional Information:** | |
| **Question** | **Response** |
| Are you submitting this manuscript to a special series or article collection? | No |
| **Experimental design and statistics**<br><br><br>Full details of the experimental design and statistical methods used should be given in the Methods section, as detailed in our Minimum Standards Reporting Checklist. Information essential to interpreting the data presented should be made available in the figure legends.<br><br><br>Have you included all the information requested in your manuscript? | Yes |
| **Resources**<br><br><br>A description of all resources used, including antibodies, cell lines, animals and software tools, with enough information to allow them to be uniquely identified, should be included in the Methods section. Authors are strongly encouraged to cite Research Resource Identifiers (RRIDs) for antibodies, model organisms and tools, where possible.<br><br><br>Have you included the information requested as detailed in our Minimum Standards Reporting Checklist? | Yes |
| **Availability of data and materials** | Yes |

*Powered by Editorial Manager® and ProduXion Manager® from Aries Systems Corporation*

| | |
|---|---|
| All datasets and code on which the conclusions of the paper rely must be either included in your submission or deposited in publicly available repositories (where available and ethically appropriate), referencing such data using a unique identifier in the references and in the "Availability of Data and Materials" section of your manuscript.<br><br>Have you have met the above requirement as detailed in our Minimum Standards Reporting Checklist? | |

Manuscript
Click here to access/download;Manuscript;manuscript.pdf
Click here to view linked References

*GigaScience*, 2018, 1–8

doi: xx.xxxx/xxxx
Manuscript in Preparation
Data Note

DATA NOTE

# Ultra-deep, long-read nanopore sequencing of mock microbial community standards

Samuel M. Nicholls[1†], Joshua C. Quick[1†], Shuiquan Tang[2] and Nicholas J. Loman[1*]

[1]Institute of Microbiology and Infection, School of Biosciences, University of Birmingham, UK and [2]Zymo Research Corporation, Irvine, California, USA

[*]n.j.loman@bham.ac.uk
[†]Contributed equally.

## Abstract

**Background**: Long sequencing reads are information-rich: aiding *de novo* assembly and reference mapping, and consequently have great potential for the study of microbial communities. However, the best approaches for analysis of long-read metagenomic data are unknown. Additionally, rigorous evaluation of bioinformatics tools is hindered by a lack of long-read data from validated samples with known composition.
**Methods**: We sequenced two commercially-available mock communities containing ten microbial species (ZymoBIOMICS Microbial Community Standards) with Oxford Nanopore GridION and PromethION. Both communities and the ten individual species isolates were also sequenced with Illumina technology.
**Data**: We generated 14 and 16 Gbp from two GridION flowcells and 150 and 153 Gbp from two PromethION flowcells for the evenly-distributed and log-distributed communities respectively. Read length N50 ranged between 5.3 Kbp and 5.4 Kbp over the four sequencing runs. Basecalls and corresponding signal data are made available (4.2 TB in total).
**Results**: Alignment to Illumina-sequenced isolates demonstrated the expected microbial species at anticipated abundances, with the limit of detection for the lowest abundance species below 50 cells (GridION). *De novo* assembly of metagenomes recovered long contiguous sequences without the need for pre-processing techniques such as binning.
**Conclusions**: We present ultra-deep, long-read nanopore datasets from a well-defined mock community. These datasets will be useful for those developing bioinformatics methods for long-read metagenomics and for the validation and comparison of current laboratory and software pipelines.

**Key words**: bioinformatics; metagenomics; mock community; nanopore; single-molecule sequencing; real-time sequencing; benchmark; GridION; PromethION; Illumina; *de novo* assembly

## Data Description

Whole-genome sequencing of microbial communities (metagenomics) has revolutionised our view of microbial evolution and diversity, with numerous potential applications for microbial ecology, clinical microbiology and industrial biotechnology [1, 2]. Typically, metagenomic studies use high-throughput sequencing platforms (*e.g.* Illumina) [3] which generate very high yield, but of limited read length (100–300 bp).

In contrast, single-molecule sequencing platforms such as the Oxford Nanopore MinION, GridION and PromethION are able to sequence very long fragments of DNA (>10 Kbp, with over 2 Mbp reported) [4, 5] and with recent improvements to the platform making metagenomic studies using nanopore more viable, such studies are increasing in frequency [6, 7, 8, 9]. Long reads help with alignment-based assignment of taxonomy and function due to their increased information content [10, 11]. Additionally, long reads permit bridg-

**Compiled on:** February 22, 2019.
Draft manuscript prepared by the author.

**Table 1.** Description of the ten organisms comprising the ZymoBIOMICS Mock Community Standards.

| Species | Type | Est. Size (Mbp) | NRRL Accession | ATCC Accession | Sequence Type | Illumina FASTQ | PacBio RSII FASTQ [17] | PacBio Sequel FASTQ [17] |
|---|---|---|---|---|---|---|---|---|
| *Bacillus subtilis* | Gram + | 4.045 | B-354 | 6633 | ST7 | ERR2935851 | SRR7498042 | SRR7415629 |
| *Cryptococcus neoformans* × *Cryptococcus deneoformans* | Yeast | 18.9 | Y-2534 | 32045 | – | ERR2935856 | – | – |
| *Enterococcus faecalis* | Gram + | 2.845 | B-537 | 7080 | ST55 | ERR2935850 | SRR7415622 | SRR7415630 |
| *Escherichia coli* | Gram – | 4.875 | B-1109 | – | ST10 | ERR2935852 | SRR7498041 | – |
| *Lactobacillus fermentum* | Gram + | 1.905 | B-1840 | 14931 | – | ERR2935857 | – | – |
| *Listeria monocytogenes* | Gram + | 2.992 | B-33116 | 19117 | ST449 | ERR2935854 | SRR7415624 | SRR7415635 |
| *Pseudomonas aeruginosa* | Gram – | 6.792 | B-3509 | 15442 | ST252 | ERR2935853 | SRR7498043 | – |
| *Saccharomyces cerevisiae* | Yeast | 12.1 | Y-567 | 9763 | – | ERR2935855 | SRR7498048 | SRR7415638 |
| *Salmonella enterica* | Gram – | 4.760 | B-4212 | – | ST139 | ERR2935848 | SRR7415626 | SRR7415636 |
| *Staphylococcus aureus* | Gram + | 2.730 | B-41012 | – | ST9 | ERR2935849 | SRR7415627 | SRR7415637 |

Table adapted from ZymoBIOMICS™ Microbial Community Standard II (Log Distribution) Instruction Manual v1.1.2 Table 2 and Appendix A. The *S. enterica* genome is listed at NRRL (B-4212) as Serovar Typhimurium LT2, but our genomic analysis shows it is likely to be Serotype Choleraesuis; indicating possible mis-annotation.

ing of repetitive sequences (within and between genomes), aiding genome completeness in *de novo* assembly [12]. However, these advantages are constrained by high error rate (≈10%), requiring the use of specific long-read alignment and assembly methods, which are either not specifically designed for metagenomics, or have not been extensively tested on real data [13].

Mock community standards are useful for the development of genomics methods [14], and for the validation of existing laboratory, software and bioinformatics approaches. For example, validating the accuracy of a taxonomic identification pipeline is important, because the consequences of erroneous taxonomic identification from a metagenomic analysis may be severe, *e.g.* in public health microbiology [15, 16] or incorrect diagnoses in clinical microbiology diagnostics. Mock community standards can also be used as positive controls during laboratory work, for example to validate that DNA extraction methods will yield expected representation of a sampled community [14].

Here, we present four nanopore sequencing datasets of two microbial community standards, providing a state-of-the-art benchmark to accelerate the development of methods for analysing long-read metagenomics data.

## Background Information

The ZymoBIOMICS Microbial Community Standards (CS and CSII) are each composed of ten microbial species: eight bacteria and two yeasts (Table 1). The organisms in CS (hereafter referred to as 'Even') are distributed equally (12%), with the exception of the two yeasts which are each present at 2%. Cell counts from organisms in CSII ('Log') community are distributed on a log scale, ranging from 89.1% (*Listeria monocytogenes*), down to 0.000089% (*Staphylococcus aureus*).

## Methods

### DNA extraction

DNA was extracted from 75 µl ZymoBIOMICS Microbial Community Standard (Product D6300, Lot ZRC190633) and 375 µl ZymoBIOMICS Microbial Community Standard II (Product D6310, Lot ZRC190842) using the ZymoBIOMICS DNA Miniprep extraction kit according to manufacturer's instructions, with the following modifications to increase fragment length and maintain the expected representation of the Gram-negative species which are already lysed in the DNA/RNA Shield storage solution. The standard was centrifuged at 8,000×*g* for 5 minutes before removing the supernatant and retaining. The cell pellet was resuspended in 750 µl lysis buffer and added to the ZR BashingBead lysis tube. Bead-beating was performed on a FastPrep-24 (MP Biomedicals) instrument for 2 cycles of 40 seconds at $6.0\,m\,s^{-1}$, with 5 minutes sitting on ice between cycles. The bead tubes were centrifuged at 10,000×*g* for 1 minute and 450 µl of supernatant was transferred to a Zymo Spin III-F filter before being centrifuged again at 8000×*g* for 1 minute. 45 µl (Even) and 225 µl (Log) of the supernatant retained earlier was combined with 450 µl filtrate before adding 1485 µl (Even) or 2025 µl (Log) Binding Buffer and mixing before loading onto the column. Methods are available via dx.doi.org/10.17504/protocols.io.x9tfr6n.

### Nanopore sequencing library preparation

Quantification steps were performed using the dsDNA HS assay for Qubit. DNA was size-selected by cleaning up with 0.45× volume of Ampure XP (Beckman Coulter) and eluted in 100 µl EB (Qiagen). Libraries were prepared from 1400 ng input DNA

**Table 2.** Summary of the four nanopore sequencing experiments.

| Signal Accession | FASTQ Accession | Sequencer | Standard (Lot) | Time (h) | Reads (M) | N50 (Kbp) | Quality (Median Q) | Yield (Gbp) | Q>7 (Gbp) |
|---|---|---|---|---|---|---|---|---|---|
| ERR2887847 | ERR3152364 | GridION | Zymo CS Even ZRC190633 | 48 | 3.49 | 5.3 | 10.3 | 14.38 | 12.39 |
| ERR2887850 | ERR3152366 | GridION | Zymo CSII Log ZRC190842 | 48 | 3.67 | 5.4 | 9.8 | 16.51 | 13.97 |
| ERR2887848<br>ERR2887849 | ERR3152365 | PromethION<br>PromethION | Zymo CS Even ZRC190633<br>Zymo CS Even ZRC190633 | 64<br>– | 35.7 | 5.4 | 10.5 | 150.88 | 130.32 |
| ERR2887851<br>ERR2887852 | ERR3152367 | PromethION<br>PromethION | Zymo CSII Log ZRC190842<br>Zymo CSII Log ZRC190842 | 64<br>– | 34.5 | 5.4 | 10.7 | 153.31 | 133.68 |

PromethION runs were restarted following the standard 64 hour protocol. The table reflects total yield across both the standard run and subsequent restarts.

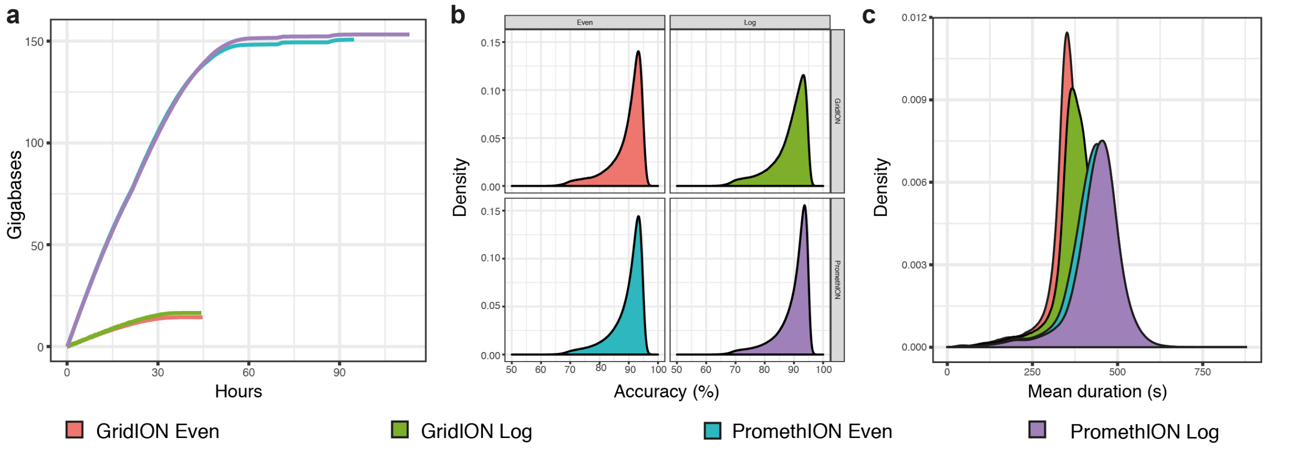


**Figure 1.** Summary plots for the four generated data sets: (a) collector's curve showing sequencing yield over time for each of the four sequencing runs, (b) density plot showing sequence accuracy (BLAST-like identities), (c) density plot showing sequencing speed over time by sequencing experiment.

using the SQK-LSK109 kit (Oxford Nanopore Technologies) as per manufacturer's protocol, except incubation times for end-repair, dA-tailing and ligation were increased to 30 minutes to improve ligation efficiency. The even and log libraries were split and used on both the GridION and PromethION flowcells.

### Sequencing

Sequencing libraries were quantified and two aliquots of 50 ng and 400 ng were prepared for GridION and PromethION sequencing respectively. The GridION sequencing was performed using FLO-MIN106 (rev.C) flowcells, MinKNOW 1.15.1 and standard 48-hour run script with active channel selection enabled. The PromethION sequencing was performed using FLO-PRO002 flowcells, MinKNOW 1.14.2 and standard 64-hour run script with active channel selection enabled.

Refuelling was performed approximately every 24h (GridION, PromethION) by loading 75 µl (GridION) or 150 µl (PromethION) refuelling mix (SQB diluted 1:1 with nuclease-free water). Additionally, after the standard scripts had completed the PromethION was restarted several times to utilise remaining active pores and maximise total yield.

### Nanopore basecalling

Reads were basecalled on-instrument using the Guppy v2.2.2 GPU basecaller (Oxford Nanopore Technologies) with the supplied dna_r9.4.1_450bps_flipflop_prom.cfg configuration (PromethION) and dna_r9.4.1_450bps_flipflop.cfg (GridION).

### Illumina sequencing

DNA was extracted from pure cultures of each species using the ZymoBIOMICS DNA Miniprep Kit. Library preparation was performed using the Kapa HyperPlus Kit with 100 ng DNA as input and TruSeq Y-adapters. The purified library derived from each sample was quantified by TapeStation (Agilent 4200) and pooled together in an equimolar fashion. The multiplexed isolates were sequenced on an Illumina HiSeq 1500 instrument using 2×101 bp (paired-end) sequencing, over four lanes. Raw reads were demultiplexed using bcl2fastq v2.17. Shotgun sequencing of the even and log communities was performed with the same protocol, with the exception that the log community was sequenced individually on two flowcell lanes, and the even community was instead sequenced on an Illumina MiSeq using 2×151 bp (paired-end) sequencing.

## Bioinformatics Methods

### Illumina draft assembly

For the purposes of estimating sequencing coverage and contiguity, we constructed a draft assembly from our available Illumina sequencing data. Illumina reads for each of the ten isolates were assembled using SPAdes v3.12.0 [18] with paired-end reads as input, using parameters -m 512 -t 12. Scaffolds

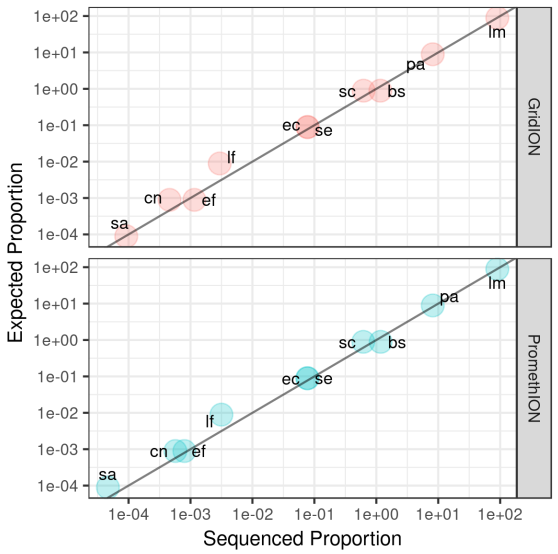


**Figure 2.** Proportion of sequenced bases assigned by minimap2 to each of the 10 organisms that were sequenced (x-axis), against the proportion of yield expected given the known composition (y-axis) of the Zymo CSII (Log) standard.

**Table 3.** Summary statistics for Illumina sequencing data.

| Dataset | Pairs (M) | Yield (Gbp) | phred≥30 (%) | Accession |
|---|---|---|---|---|
| Isolates | 13.53 ± 5.23 | 2.73 ± 1.06 | 87.72% ± 5.43% | see Table 1 |
| CS (Even) | 8.8 | 2.65 | 95.12 % | ERR2984773 |
| CSII (Log) | 47.8 | 9.66 | 95.71 % | ERR2935805 |

Illumina sequencing was performed on an Illumina HiSeq 1500, with the exception of the even community which was sequenced on an Illumina MiSeq.

**Table 4.** Read alignment statistics for even samples, showing absolute measurements and proportion of sequencing yield and the estimated genome coverage obtained for each organism in the mock community.

| | | GridION | | | | PromethION | | | |
|---|---|---|---|---|---|---|---|---|---|
| Species | Expected Proportion | Yield (Gbp) | Measured Proportion | Aln. N50 (Kbp) | Coverage ($\times$) | Yield (Gbp) | Measured Proportion | Aln. N50 (Kbp) | Coverage ($\times$) |
| *Bacillus subtilis* | 12 | 2.12 | 19.32 | 4.30 | 524.51 | 21.55 | 19.02 | 4.40 | 5326.44 |
| *Listeria monocytogenes* | 12 | 1.60 | 14.56 | 4.47 | 534.26 | 16.23 | 14.33 | 4.58 | 5424.46 |
| *Enterococcus faecalis* | 12 | 1.34 | 12.24 | 4.45 | 472.47 | 13.67 | 12.07 | 4.57 | 4805.60 |
| *Staphylococcus aureus* | 12 | 1.24 | 11.28 | 4.47 | 453.84 | 12.59 | 11.11 | 4.59 | 4611.61 |
| *Salmonella enterica* | 12 | 1.10 | 9.99 | 8.55 | 230.51 | 11.69 | 10.32 | 8.95 | 2456.19 |
| *Escherichia coli* | 12 | 1.09 | 9.93 | 8.31 | 223.59 | 11.62 | 10.26 | 8.71 | 2382.59 |
| *Pseudomonas aeruginosa* | 12 | 1.07 | 9.70 | 8.98 | 156.85 | 11.45 | 10.11 | 9.38 | 1686.34 |
| *Lactobacillus fermentum* | 12 | 1.02 | 9.28 | 3.62 | 534.73 | 10.34 | 9.13 | 3.73 | 5425.69 |
| *Saccharomyces cerevisiae* | 2 | 0.21 | 1.92 | 4.09 | 17.46 | 2.12 | 1.87 | 4.18 | 175.23 |
| *Cryptococcus neoformans* | 2 | 0.20 | 1.78 | 4.45 | 10.37 | 2.00 | 1.77 | 4.54 | 105.82 |

**Table 5.** Read alignment statistics for log samples, describing sequencing yield and estimated genome coverage obtained for each organism in the mock community.

| | GridION | | | PromethION | | |
|---|---|---|---|---|---|---|
| Species | Yield (Gbp) | Aln. N50 (Kbp) | Coverage ($\times$) | Yield (Gbp) | Aln. N50 (Kbp) | Coverage ($\times$) |
| *Listeria monocytogenes* | 12.10 | 4.95 | 4043.90 | 110.09 | 4.97 | 36 796.21 |
| *Pseudomonas aeruginosa* | 1.10 | 9.38 | 161.45 | 9.99 | 9.33 | 1471.41 |
| *Bacillus subtilis* | 0.16 | 5.03 | 38.67 | 1.44 | 5.04 | 356.00 |
| *Saccharomyces cerevisiae* | 0.08 | 4.78 | 6.93 | 0.75 | 4.75 | 62.33 |
| *Salmonella enterica* | 0.01 | 9.20 | 2.20 | 0.10 | 9.17 | 20.04 |
| *Escherichia coli* | 0.01 | 8.65 | 2.14 | 0.09 | 9.17 | 19.24 |
| *Lactobacillus fermentum* | $4 \times 10^{-4}$ | 3.40 | 0.210 | 0.004 | 3.37 | 2.03 |
| *Enterococcus faecalis* | $2 \times 10^{-4}$ | 7.62 | 0.055 | $1 \times 10^{-3}$ | 6.05 | 0.34 |
| *Cryptococcus neoformans* | $6 \times 10^{-5}$ | 4.41 | 0.003 | $7 \times 10^{-4}$ | 4.97 | 0.037 |
| *Staphylococcus aureus* | $1 \times 10^{-5}$ | 7.12 | 0.005 | $5 \times 10^{-5}$ | 3.58 | 0.020 |

Note that expected and measured proportions are illustrated by Figure 2.

from `SPAdes` less than 500 bp length or with less than 10$\times$ coverage were removed. The remaining scaffolds were combined into a single mock community draft assembly for downstream analysis. Multilocus sequence typing (MLST) of the scaffolds was conducted with `mlst` (https://github.com/tseemann/mlst).

## PacBio draft assembly

A recently released orthogonal data set from McIntyre *et al.* (2019) includes individual PacBio sequencing of eight of the ten organisms that compose the two Zymo communities [17]. Assemblies for the eight isolates that passed quality control (excluding *L. fermentum* and *C. neoformans*) were generated with `HGAP v2` [19]. Assemblies have been made available by the authors and were downloaded from https://github.com/al-mcintyre/mCaller_analysis_scripts/tree/master/assemblies (`Git` commit `dba494d`) for the purposes of assessing metagenomic assembly accuracy for the 7 bacterial species where complete genomes were available.

## Sequencing coverage estimation

Nanopore reads were aligned to the Illumina draft assembly using `minimap2` [20] v2.14-r883 with parameters `-ax map-ont -t 12` and converted to a sorted BAM file using `samtools` [21]. To reduce erroneous mappings, alignment BAM files were filtered using a script `bamstats.py` according to the following criteria; reference mapping length ≥500 bp, map quality (MAPQ) > 0, there are no supplementary alignments for this read and read is not a secondary alignment. Per-species coverage summary statistics were generated using the `summariseStats.R` Rscript.

## Nanopore read accuracy

Read accuracy was determined by calculating `BLAST`-like identities from the filtered alignments (as per http://lh3.github.io/2018/11/25/on-the-definition-of-sequence-identity), calculated as $(L - NM)/L$ using the `minimap2` number of mismatches (`NM`) SAM tag and the sum of match, insertion and deletion CIGAR operations (`L`).

## Metagenomic assembly and contiguity estimation

Metagenomic assemblies were constructed with `wtdbg2` v2.2 [22] from the nanopore sequencing of the communities. `wtdbg2` was compiled from source via `Git` commit `904f2b3`. For GridION, all nanopore reads were used. For PromethION, a 25% subsample was selected with `seqtk` (https://github.com/lh3/seqtk).

Assemblies were conducted under a variety of parameter values for homopolymer-compressed k-mer size (`-p`), minimum graph edge weight support (`-e`) and read length threshold (`-L`). Global parameters for all runs (`-S1 -K10000 --node-max 6000`) were used to turn-off k-mer subsampling (to remove assembly stochasticity) and increase the coverage thresholds applied to k-mers and constructed nodes.

Assembled contigs were assigned to taxa with `kraken2` [23] (`--use-names -t12`) using a database containing all of the archaeal, bacterial, fungal, protozoal and viral sequences from `RefSeq`, and `UniVec_Core` (database download links are in our repository). The `kraken2` output was parsed with `extracken.py` and plotted with `contiguity.R` to visually assess contiguity. Following assignment, contigs can be extracted into separate FASTA with `extract_contigs_with_kraken.py`.

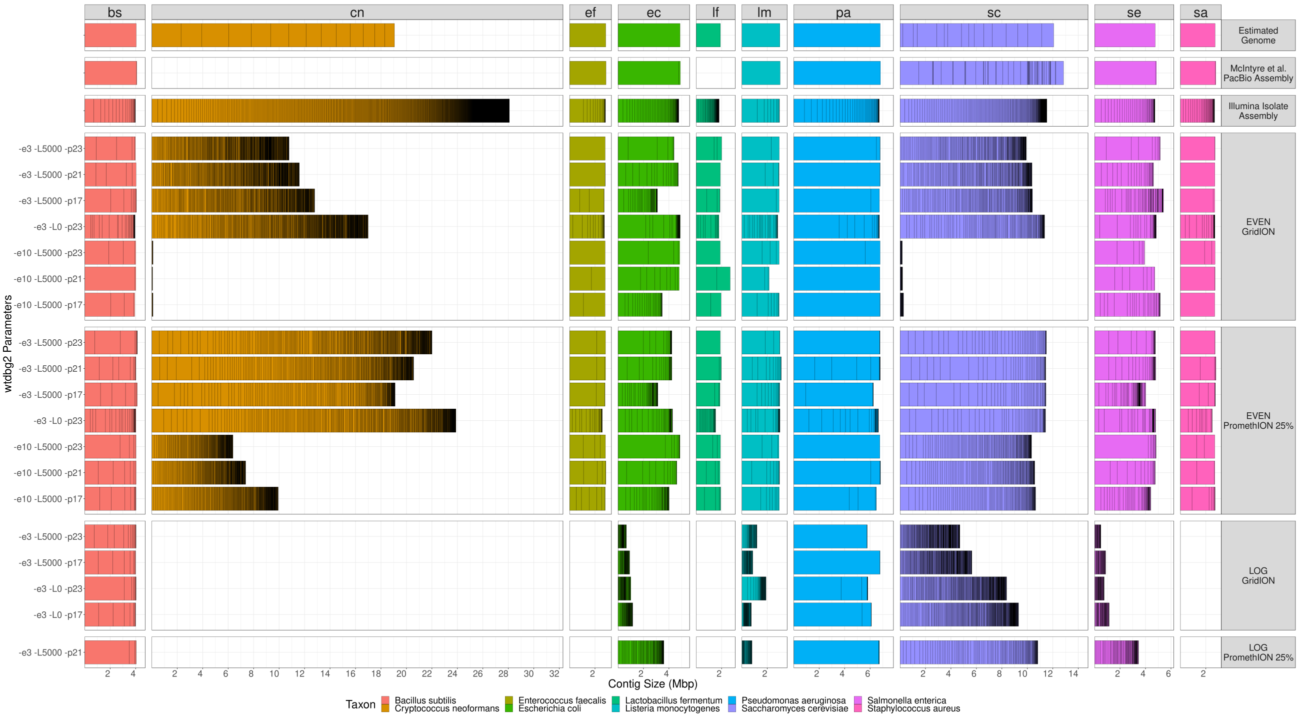


**Figure 3.** Bar plots demonstrating total length and contiguity of genomic assemblies obtained with `wtdbg2` from each of the long-read nanopore data sets. For each organism in the community (coloured columns), contigs longer than 10 kbp are horizontally stacked along the x-axis. Each row represents a run of `wtdbg2`, with the parameters for edge support, read length threshold and homopolymer-compressed k-mer size labelled on the left. Assemblies are grouped by the data set on which they were run (row facets). Additionally, assemblies may be compared to the estimated true genome size, the available McIntyre *et al.* PacBio assemblies, and per-isolate Illumina `SPAdes` assembly. Estimated genomes sizes are the same as those found in Table 1, however to display approximate chromosomes, the two yeasts were replaced by their corresponding canonical NCBI references for visualisation purposes only. The *C. neoformans* strain used by the Zymo standards is a diploid genetic cross, which may explain the larger assemblies, compared to the represented estimated haploid size.

## Assembly polishing

After inspection of the `contiguity.R` plot, eight high-contiguity assemblies were selected for polishing. Polishing consisted of two iterations of `racon` [24], followed by `medaka` (https://github.com/nanoporetech/medaka) and two iterations of `pilon` [25]. `racon` v1.3.2 was used to polish contigs with the nanopore reads. `medaka` v0.5.0 was used to polish the `racon` polished contigs, with the nanopore reads specifying the `r941_flip` model. The PromethION assemblies were polished using the same `seqtk`-derived 25% subset from which the assemblies were constructed. `pilon` v1.23 was used to polish the `medaka` polished contigs, with the CS (Even) community Illumina reads.

## Estimation of genome completeness

To estimate accuracy of the polished assemblies, contigs were first assigned to taxa and extracted into separate FASTA using `kraken2` as previously described. For the seven bacteria for which corresponding PacBio draft assemblies were available, sequence identity dotplots were generated using a modified version of `minidot` (https://github.com/SamStudio8/minidot) which uses `minimap2` (`-x asm10 --no-long-join --dual=yes -P`) to align the polished contigs binned by `kraken2`, to the corresponding PacBio draft. Genome completeness was estimated with `CheckM` v1.0.13 [26] using the `taxonomy_wf` subcommand, after each phase of the polishing pipeline. `CheckM` was executed separately for each `kraken2` bin that had a corresponding PacBio reference, specifying the appropriate species for the bin to `taxonomy_wf`. We report the `CheckM` "Completeness" score, which estimates completeness by identifying collocated marker gene sets on the assembled contigs as a proportion of the total collection of marker gene sets expected for a specific taxon.

# Results

## Nanopore sequencing metrics

We generated a total of 335.1 Gbp of sequence from the four nanopore sequencing runs (Table 2, Figure 1a). PromethION flowcells generated approximately ten-times more sequencing data than the comparative GridION runs and showed equivalent read length N50 and read accuracy (Figure 1b). We observe a difference in sequencing speed between the PromethION (mean 419 bps and 437 bps for even and log) and the GridION (mean speed 352 and 372 bps for even and log) (Figure 1c).

## Illumina sequencing metrics

Illumina datasets for the ten individually sequenced isolates averaged 13.53 million pairs of reads (ranging between 7.1 – 23.2 million), with proportions of reads with a mean `phred` score ≥30 ranging between 75.51% – 93.09% (Table 3). Illumina sequencing generated 8.8 million pairs of reads (2×151 bp, MiSeq), and 47.8 million pairs of reads (2×101 bp, HiSeq) for the even and log community, respectively (Table 3).

## Nanopore mapping statistics

We identify the presence of all 10 microbial species in the community, for both even and log samples, in expected proportions (Figure 2). For the even community, the GridION results provide sufficient depth (i.e. ≫ 30× coverage) to potentially assemble all eight of the bacteria. The coverage of the yeast genomes were lower (10× and 17×), potentially sufficient for assembly scaffolding. On the PromethION all genomes had >100× mean coverage (Tables 4 and 5).

**Table 6.** Sequence identity dotplots and `CheckM` genome completeness scores for each of the seven bacteria for which there was a corresponding PacBio assembly from McIntyre *et al.* (2019). Four `wtdbg2` assembly conditions are represented, varying the homopolymer-compressed k-mer parameter '`p`' and the graph minimum edge weight threshold '`e`'. The read length threshold '`L`' was fixed at 5000 bp. The left and right halves of the table correspond to the same assembly condition for the GridION and 25% PromethION sequencing data, respectively. The L50/L95 refers to the number of assembled contigs required to span at least 50% and 95% of the estimated genome size (see Table 1). An – indicates that the set of assembled contigs assigned to a taxon were not of sufficient total length to cover 95% of the estimated size. `CheckM` genome completeness scores are expressed as a percentage and were calculated per-organism at the end of each polishing phase.

| GridION | | | | | | | | PromethION | | | | | | |
|---|---|---|---|---|---|---|---|---|---|---|---|---|---|---|
| bs | ef | ec | lm | pa | se | sa | Assembly | bs | ef | ec | lm | pa | se | sa |
| 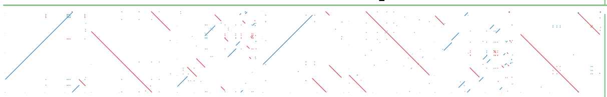 | | | | | | | -p 21 -e 3 | 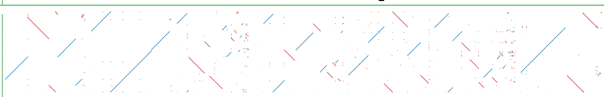 | | | | | | |
| 2 / 5 | 1 / 1 | 5 / 17 | 1 / 3 | 1 / 1 | 6 / 21 | 1 / 1 | L50/L95 | 2 / 5 | 1 / 2 | 4 / – | 3 / 8 | 2 / 5 | 4 / 15 | 1 / 2 |
| 74.27 | 76.10 | 70.14 | 76.11 | 78.64 | 66.78 | 78.48 | Base | 70.25 | 72.82 | 62.02 | 69.46 | 78.17 | 65.01 | 74.35 |
| 86.65 | 88.08 | 84.21 | 83.41 | 92.96 | 82.74 | 90.27 | +Racon×2 | 83.20 | 83.93 | 71.02 | 82.33 | 90.38 | 77.68 | 86.97 |
| 97.45 | 99.07 | 94.46 | 97.50 | 97.33 | 95.10 | 98.35 | +Medaka | 95.83 | 97.74 | 81.65 | 96.70 | 99.14 | 91.48 | 97.69 |
| 98.42 | 99.66 | 95.46 | 98.57 | 99.77 | 96.98 | 98.88 | +Pilon×2 | 98.44 | 99.66 | 83.59 | 98.66 | 99.73 | 95.02 | 98.20 |
| 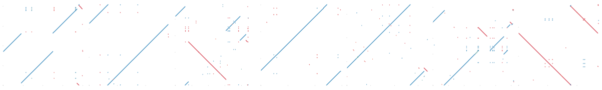 | | | | | | | -p 23 -e 3 | 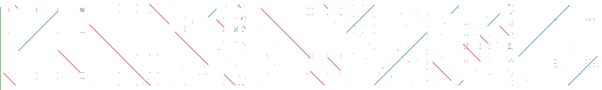 | | | | | | |
| 2 / 4 | 1 / 1 | 2 / – | 1 / 2 | 1 / 1 | 2 / 4 | 1 / 1 | L50/L95 | 2 / 3 | 1 / 2 | 2 / – | 1 / 3 | 1 / 1 | 3 / 7 | 1 / 1 |
| 73.42 | 75.81 | 67.44 | 74.44 | 81.08 | 70.54 | 78.20 | Base | 68.20 | 71.32 | 59.15 | 70.02 | 79.85 | 65.62 | 74.04 |
| 84.81 | 87.30 | 79.83 | 84.57 | 92.53 | 85.83 | 88.64 | +Racon×2 | 83.23 | 83.36 | 67.16 | 82.42 | 87.72 | 77.46 | 84.17 |
| 96.60 | 98.80 | 89.41 | 98.06 | 98.04 | 97.46 | 98.23 | +Medaka | 95.98 | 97.71 | 79.47 | 97.42 | 99.03 | 94.14 | 97.90 |
| 97.83 | 99.66 | 90.36 | 99.15 | 99.82 | 98.67 | 98.88 | +Pilon×2 | 98.34 | 99.66 | 81.65 | 99.27 | 99.77 | 98.18 | 98.88 |
| 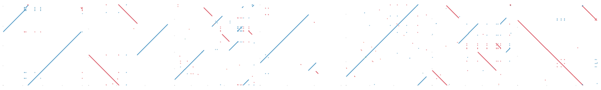 | | | | | | | -p 21 -e 10 | 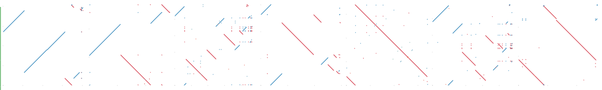 | | | | | | |
| 1 / 1 | 1 / 2 | 3 / 8 | 1 / – | 1 / 1 | 2 / 7 | 1 / 1 | L50/L95 | 2 / 5 | 2 / 4 | 3 / 14 | 2 / 7 | 1 / 2 | 3 / 10 | 1 / 2 |
| 74.29 | 76.07 | 72.21 | 57.20 | 79.64 | 68.99 | 78.28 | Base | 70.82 | 72.29 | 67.56 | 71.91 | 79.04 | 66.33 | 74.20 |
| 85.36 | 86.11 | 84.53 | 62.94 | 92.28 | 84.46 | 90.46 | +Racon×2 | 83.88 | 85.62 | 77.20 | 84.26 | 90.35 | 79.43 | 88.35 |
| 97.14 | 99.11 | 95.95 | 71.21 | 98.06 | 96.57 | 98.55 | +Medaka | 96.87 | 97.57 | 90.88 | 97.43 | 98.92 | 95.51 | 98.01 |
| 98.27 | 99.66 | 97.17 | 72.24 | 99.53 | 98.34 | 98.78 | +Pilon×2 | 98.43 | 99.66 | 92.58 | 99.15 | 99.72 | 97.85 | 98.86 |
| 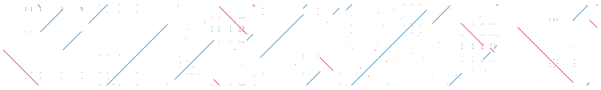 | | | | | | | -p 23 -e 10 | 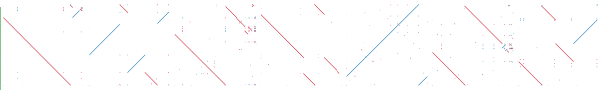 | | | | | | |
| 2 / 3 | 1 / 1 | 2 / 3 | 1 / 4 | 1 / 2 | 2 / – | 1 / 3 | L50/L95 | 1 / 3 | 2 / 4 | 1 / 4 | 1 / 4 | 1 / 1 | 1 / 4 | 2 / 3 |
| 73.71 | 77.52 | 73.73 | 75.42 | 82.06 | 60.74 | 79.24 | Base | 69.27 | 71.85 | 70.35 | 71.17 | 80.41 | 67.06 | 74.98 |
| 86.42 | 88.31 | 84.69 | 85.14 | 92.81 | 71.29 | 87.81 | +Racon×2 | 81.55 | 84.89 | 82.92 | 83.81 | 89.17 | 80.33 | 88.61 |
| 97.16 | 98.83 | 94.26 | 96.86 | 97.86 | 82.42 | 98.45 | +Medaka | 96.62 | 98.46 | 95.42 | 96.40 | 98.72 | 96.26 | 97.74 |
| 98.44 | 99.66 | 98.13 | 98.69 | 99.83 | 83.58 | 98.86 | +Pilon×2 | 98.33 | 99.66 | 97.14 | 98.69 | 99.72 | 98.75 | 98.78 |
| bs | ef | ec | lm | pa | se | sa | | bs | ef | ec | lm | pa | se | sa |

For the log-distributed community, three taxa have sufficient coverage for assembly on GridION, compared with four on PromethION. On PromethION, a further two genomes (*S. enterica* and *E. coli*) have sufficient coverage for assembly scaffolding. We are able to detect *S. aureus*, the lowest abundance organism on both platforms, with 19 reads from PromethION (from 400 cell input) and 4 reads from GridION (from 50 cell input).

## Nanopore metagenomic assemblies

We assessed the contiguity of our nanopore metagenomic assemblies for each run with different assembly parameters.

For the even community, genomes of the expected size were present for each of the bacterial species, contained in small numbers of large contigs (Figure 3). However, the two yeasts are highly fragmented, consistent with their low read depth.

*L. monocytogenes* is poorly assembled in the log dataset despite being the most abundant organism, indicating very high sequence coverage may be detrimental to the performance of `wtdbg2`. We note that assembling the entire PromethION dataset resulted in less complete and more fragmented assemblies. This led us to random subsample the PromethION data to 25% of the total dataset which improved the assembly results.

After subsampling, assemblies of the even community from the GridION and PromethION are similar. However, the assemblies from PromethION data had better representation of the yeasts in terms of size and contiguity (particularly for *C. neoformans*), likely due to the higher coverage of these species.

We also assessed the completeness of polished genomes for a selection of our highly-contiguous metagenomic assemblies.

For the GridION, we observe for at least one of the polished assemblies, four bacterial genomes are reconstructed to at least 95% of their length (L95) in a single contig. For PromethION, we observe that for seven bacteria, at least half the genome (L50) is reconstructed on a single contig, for at least one assembly condition (Table 6).

Genome completeness as estimated by `CheckM` averaged 73.95% and 70.98% over the four unpolished assemblies, for the GridION and PromethION respectively. We observed each phase of the polishing pipeline improved completeness. For the GridION assemblies, completeness was incrementally improved by 11.57 pp, 10.14 pp and 1.25 pp for two iterations of `racon`, one iteration of `medaka` and two iterations of short read polishing with `pilon`, respectively. For the PromethION, the three polishing phases incrementally improved assemblies by an average of 11.92 pp, 12.69 pp and 1.77 pp. In almost all cases, polishing yields near complete genomes ($\geq$90%) genomes.

## Discussion

There are several noteworthy aspects of this dataset: We generated over 300 Gbp of sequence data from the Oxford Nanopore PromethION and 30 gigabases from the Oxford Nanopore GridION, on a well-characterised mock community sample and we have made basecalls and electrical signal data for each of the four runs presented here available: a combined dataset size of over four terabytes. The availability of the raw signal permits future basecalling of the data (an area under rapid development), as well as signal-level polishing and the detection of methylated bases [27].

Individual sequencing libraries were split between the GridION and PromethION, permitting direct comparisons of the instruments to be made. We observed high concordance between the datasets from each platform. We note the sequencing speed of the PromethION is faster than the GridION, which we attribute to different running temperatures on these instruments (39°C versus 34°C, respectively).

Confident detection of *S. aureus* was demonstrated for the GridION run to <50-cells using the log community. The PromethION generated around five times more *S. aureus* reads as the GridION, however we loaded eight times as much library, making it appear less sensitive. It may be possible to reduce the input to PromethION flowcells, but we have not attempted this.

Early results of metagenomic assembly show promise for reconstruction of whole microbial genomes from mixed samples without a binning step. We focused on the developing `wtdbg2` software as the established `minimap2` and `miniasm` method resulted in excessively large intermediate files (tens of terabases per analysis) which were impractical to store and analyse.

For the even community, using `wtdgb2` with varying parameter choices, we were able to assemble four of the bacteria into single contigs. However, no single parameter set was found to be optimum for both total genome size and contig length. Increasing `-e` improved contiguity for the even community, however this resulted in the loss of yeasts from the assembly. Increasing the read length threshold (`-L`) improved contiguity for all sample and platform combinations, at the cost of genome size. Increasing the homopolymer-compressed k-mer size (`-p`) from the default of 21 to 23 also appears to improve contiguity.

We found that `wtdbg2` expects a maximum of 200× sample coverage, and discards sequence k-mers and *de Bruijn* graph nodes with more than 200× support. Although these limits can be lifted by specifying higher `-K` and `-node-max`, we still observe more fragmented assemblies on the PromethION data (especially for the 100% PromethION data [not shown]) potentially indicating a need to further tune the algorithm to account for the large differences in coverage between genomes. It should be noted that `wtdbg2` is still under active development, making it difficult to make concrete recommendations for parameters.

We found that any form of polishing improves the completeness of assemblies, likely due to the correction of frameshifts caused by indels. Short read polishing with `pilon` also improves the assemblies, despite low coverage of the Illumina even community data and the results might be expected to improve further with increased coverage.

The availability of this dataset should help with further improvements to long-read assembly techniques.

Other mock microbial samples are available which we did not test here. A notable alternative mock community sample is from the Human Microbiome Project (HMP) and consists of 20 microbial samples (available from BEI Resources). This mock community have been sequenced as part of other studies, although the datasets are much smaller than the ones presented here [9, 28]. Bertrand *et al*. presented a synthetic mock community of their own construction to demonstrate hybrid nanopore-Illumina metagenome assemblies [12].

### Re-use potential

The provision of Illumina reads for each isolate permits a ground-truth to be obtained for the individual species contained in the mock community. This will be useful for training new nanopore basecalling and polishing models, long-read aligners, variant callers, and validating taxonomic assignment and assembly software and pipelines.

## Availability of source code and requirements

Python and R scripts used to generate the summary information and analyses are open source and freely available via our repository (https://github.com/LomanLab/mockcommunity), under the MIT license. Our pipeline was orchestrated with `Snakemake` [29], the workflow is available from our repository.

## Availability of supporting data and materials

This manuscript, and its supporting data are available under a Creative Commons Attribution 4.0 International license.

Unprocessed FASTQ from the Illumina sequencing of the ten isolates are available at the European Nucleotide Archive, via the identifiers listed in Table 1, identifiers for the even and log community Illumina sequencing can be found in Table 3.

Both the raw signal, and basecalled FASTQ for our nanopore sequencing experiments are available at the European Nucleotide Archive, via the identifiers listed in Table 2.

The `SPAdes`-assembled Illumina draft reference, and the collection of nanopore assemblies for each `wtdbg2` condition are linked to from our GitHub repository (https://github.com/LomanLab/mockcommunity), along with the `kraken2` database used for taxonomic classification of the assembled contigs.

Further updates (such as updated references, or new assemblies) will be made available through our project website https://lomanlab.github.io/mockcommunity/.

## Declarations

### Consent for publication

Not applicable

### Competing Interests

Cambridge Biosciences provided the ZymoBIOMICS Microbial Community Standard free of charge. ST is an employee of Zymo Research Corporation. NJ has received Oxford Nanopore Technologies (ONT) reagents free of charge to support his research programme. NJ and JQ have received travel expenses to speak at ONT events. NL has received an honorarium to speak at an ONT company meeting.

### Funding

SN is funded by the Medical Research Foundation and the NIHR STOP-COLITIS project. JQ is funded by the NIHR Surgical Reconstruction and Microbiology Research Centre. The NIHR SRMRC is a partnership between The National Institute for Health Research, University Hospitals Birmingham NHS Foundation Trust, the University of Birmingham, and the Royal Centre for Defence Medicine. NL is funded by an MRC Fellowship in Microbial Bioinformatics under the CLIMB project.

## Author's Contributions

Conceptualization: NL, Methodology: NL JQ SN ST, Software: SN NL, Validation: SN NL, Formal analysis: SN NL, Investigation: NL JQ SN, Resources: NL ST, Data Curation: SN NL ST, Writing – original draft preparation: SN, Writing – review and editing: SN NL JQ ST, Visualization: SN NL, Supervision: NL, Project administration: NL, Funding acquisition: NL ST

## Acknowledgements

We are grateful to Radoslaw Poplawski (University of Birmingham) for assistance with CLIMB virtual machines and file systems to support this research. We thank Divya Mirrington (Oxford Nanopore Technologies) for advice on PromethION library preparation and sequencing. We thank Hannah McDonnell at Cambridge Biosciences for providing the ZymoBIOMICS Microbial Community Standards. We thank Jared Simpson (Ontario Institute for Cancer Research), Matt Loose (University of Nottingham) and John Tyson (University of British Columbia) for useful discussions and advice. We thank Christopher Mason and Alexa McIntyre (Cornell University) for making PacBio data available ahead of publication.

## References

1. Handelsman J. Metagenomics: application of genomics to uncultured microorganisms. Microbiology and Molecular Biology Reviews 2004 Dec;68(4):669–685.
2. Hug LA, Baker BJ, Anantharaman K, Brown CT, Probst AJ, Castelle CJ, et al. A new view of the tree of life. Nature Microbiology 2016 Apr;1:16048.
3. Quince C, Walker AW, Simpson JT, Loman NJ, Segata N. Shotgun metagenomics, from sampling to analysis. Nature Biotechnology 2017 Sep;35(9):833–844.
4. Jain M, Koren S, Miga KH, Quick J, Rand AC, Sasani TA, et al. Nanopore sequencing and assembly of a human genome with ultra-long reads. Nature Biotechnology 2018 Jan;36:338.
5. Payne A, Holmes N, Rakyan V, Loose M. BulkVis: a graphical viewer for Oxford nanopore bulk FAST5 files. Bioinformatics 2018;p. bty841.
6. Sanderson ND, Street TL, Foster D, Swann J, Atkins BL, Brent AJ, et al. Real-time analysis of nanopore-based metagenomic sequencing from infected orthopaedic devices. BMC Genomics 2018 Sep;19(1):714.
7. Charalampous T, Richardson H, Kay GL, Baldan R, Jeanes C, Rae D, et al. Rapid Diagnosis of Lower Respiratory Infection using Nanopore-based Clinical Metagenomics. bioRxiv 2018;p. 387548.
8. Somerville V, Lutz S, Schmid M, Frei D, Moser A, Irmler S, et al. Long read-based de novo assembly of low complex metagenome samples results in finished genomes and reveals insights into strain diversity and an active phage system. bioRxiv 2018;p. 476747.
9. Leggett RM, Alcon-Giner C, Heavens D, Caim S, Brook TC, Kujawska M, et al. Rapid profiling of the preterm infant gut microbiota using nanopore sequencing aids pathogen diagnostics. bioRxiv 2018;p. 180406.
10. Huson DH, Auch AF, Qi J, Schuster SC. MEGAN analysis of metagenomic data. Genome Research 2007 Mar;17(3):377–386.
11. Wommack KE, Bhavsar J, Ravel J. Metagenomics: read length matters. Applied and Environmental Microbiology 2008 Mar;74(5):1453–1463.
12. Bertrand D, Shaw J, Narayan M, Ng HQA, Kumar S, Li C, et al. Nanopore sequencing enables high-resolution analysis of resistance determinants and mobile elements in the human gut microbiome. bioRxiv 2018;p. 456905.
13. Sczyrba A, Hofmann P, Belmann P, Koslicki D, Janssen S, Dröge J, et al. Critical assessment of metagenome interpretation—a benchmark of metagenomics software. Nature Methods 2017;14(11):1063.
14. Mason CE, Afshinnekoo E, Tighe S, Wu S, Levy S. International Standards for Genomes, Transcriptomes, and Metagenomes. Journal of Biomolecular Techniques 2017 Apr;28(1):8–18.
15. Ackelsberg J, Rakeman J, Hughes S, Petersen J, Mead P, Schriefer M, et al. Lack of Evidence for Plague or Anthrax on the New York City Subway. Cell Systems 2015 Jul;1(1):4–5.
16. McIntyre AB, Ounit R, Afshinnekoo E, Prill RJ, Hénaff E, Alexander N, et al. Comprehensive benchmarking and ensemble approaches for metagenomic classifiers. Genome biology 2017;18(1):182.
17. McIntyre AB, Alexander N, Grigorev K, Bezdan D, Sichtig H, Chiu CY, et al. Single-molecule sequencing detection of N 6-methyladenine in microbial reference materials. Nature Communications 2019;10(1):579.
18. Bankevich A, Nurk S, Antipov D, Gurevich AA, Dvorkin M, Kulikov AS, et al. SPAdes: a new genome assembly algorithm and its applications to single-cell sequencing. Journal of Computational Biology 2012 May;19(5):455–477.
19. Chin CS, Alexander DH, Marks P, Klammer AA, Drake J, Heiner C, et al. Nonhybrid, finished microbial genome assemblies from long-read SMRT sequencing data. Nature Methods 2013;10(6):563.
20. Li H. Minimap2: pairwise alignment for nucleotide sequences. Bioinformatics 2018;1:7.
21. Li H, Handsaker B, Wysoker A, Fennell T, Ruan J, Homer N, et al. The sequence alignment/map format and SAMtools. Bioinformatics 2009;25(16):2078–2079.
22. Ruan J, Li H. Fast and accurate long-read assembly with wtdbg2. BioRxiv 2019;p. 530972.
23. Wood DE, Salzberg SL. Kraken: ultrafast metagenomic sequence classification using exact alignments. Genome Biology 2014 Mar;15(3):R46.
24. Vaser R, Sović I, Nagarajan N, Šikić M. Fast and accurate de novo genome assembly from long uncorrected reads. Genome Research 2017;27(5):737–746.
25. Walker BJ, Abeel T, Shea T, Priest M, Abouelliel A, Sakthikumar S, et al. Pilon: an integrated tool for comprehensive microbial variant detection and genome assembly improvement. PloS One 2014;9(11):e112963.
26. Parks DH, Imelfort M, Skennerton CT, Hugenholtz P, Tyson GW. CheckM: assessing the quality of microbial genomes recovered from isolates, single cells, and metagenomes. Genome Research 2015;25(7):1043–1055.
27. Simpson JT, Workman RE, Zuzarte PC, David M, Dursi LJ, Timp W. Detecting DNA cytosine methylation using nanopore sequencing. Nature Methods 2017 Apr;14(4):407–410.
28. Huson DH, Albrecht B, Bağcı C, Bessarab I, Górska A, Jolic D, et al. MEGAN-LR: new algorithms allow accurate binning and easy interactive exploration of metagenomic long reads and contigs. Biology Direct 2018 Apr;13(1):6.
29. Köster J, Rahmann S. Snakemake—a scalable bioinformatics workflow engine. Bioinformatics 2012;28(19):2520–2522.

 Click here to access/download;Personal Cover;Nicholls_et_al-GigaScienceResponseFeb19.pdf

Dear Editor,
Since our previous submission, our manuscript has been updated. In particular we have re-performed basecalling on the original nanopore data with an updated basecaller. Additionally, we have made comparisons between our long-read assemblies and some recently released PacBio assemblies of individuals from the same mock community. We believe this addresses the primary concern of our reviewers.

We provide detailed responses to the specific comments of our reviewers below.

---

## Reviewer 1

**R1.1.** It seems that species assignment was done in two ways, one by using Kraken on the contigs (with a database of many bacterial/viral/fungal genomes) ; and also by mapping the reads directly to the illumina assemblies of the isolates in the mixture. It would be useful to be clearer in the results which approach was used in reporting the results.

E.g. the sentence "We identify the presence of all 10 microbial species in the community, for both even and log samples, in expected proportions (Figure 2)." presumably relates to the analysis just mapping to the draft illumina assemblies?

**Response 1.1**: We agree that the manuscript could be clearer in this regard. The read-level analysis identified taxa by mapping to a combined Illumina assembly of all the isolates. The assembly-level analysis used kraken2 to assign contigs to species. We have clarified our Methods; in particular, the “Bioinformatics” subsection of the Methods has been elevated to a section of its own, allowing us to add suitable subheadings to better delimit our computational methods.

**R1.2.** Also, It seems a little surprising that there were no false positive identification of species not present in the mixture. Is this because this analysis is based on mapping to the draft illumina isolate assemblies only (see previous comment). Or, if based on kraken assignment of contigs, perhaps repetitive and/or short contigs were filtered out?

**Response 1.2**: The reviewer is correct that this is because analysis is based on mapping to the Illumina assemblies only with minimap2. If kraken2 had been used on individual reads, some level of misassignment would be expected.

**R1.3.** Could the authors present more statistics on the quality of the nanopore metagenomic assemblies, including the presence of misassemblies, any chimeric contigs, checkM completeness results; indel errors, mismatch errors, etc.

**Response 1.3**: We thank the reviewer for this helpful suggestion. In our initial manuscript this was difficult because of the absence of high quality whole-genome assemblies that would permit orthogonal assessment of assembly correctness. However, in the past weeks, PacBio assemblies of 7 out of 8 bacteria present in the Zymo community (excluding *Lactobacillus fermentum*) have been made available in McIntyre et al. Nature Communications 2019. We were able to use these genomes to assess correctness of our assemblies. Although PacBio draft assemblies are available also for *S. cerevisiae* (but not *C. neoformans*), we did not include it in the comparison because they were still heavily fragmented.

We have updated the manuscript with extensive new results (Table 6). This table demonstrates dotplots of our assembled metagenomic contigs to these new PacBio references, permitting assessment of misassemblies. Additionally, Table 6 demonstrates the CheckM completeness score for the 7 bacteria that have a corresponding PacBio assembly. Because the CheckM score is heavily contingent on consensus-level sequence identity and reflects presence of indel errors, we have included completeness results for common polishing pipelines: Racon (x2), plus Medaka, plus Illumina polishing with Pilon (x2).

**R1.4.** Also, can the authors confirm that the assemblies were done on the full nanopore dataset (rather than, for example, on each isolate separately after mapping the reads to each isolate draft illumina assembly).

**Response 1.4**: We can confirm that the assemblies are constructed from nanopore sequencing of the entire community (using 100% of GridION data, and a random 25% subsampling of PromethION sequence data). No read binning or pre-assignment steps were employed. We hope that our changes described in Response 1.1 have clarified the manuscript for readers.

**R1.5.** The authors write: "For the even community, using wtdgb2 with varying parameter choices, we were able to assemble seven of the bacteria into single contigs." , however this does not seem to be borne out by figure 3? I could only see 4 species with at least one single contig assembly. Perhaps the authors could spell out which species have a single contig assembly?

**Response 1.5**: Our assessments were initially conducted by inspecting the contiguity visualisation (Figure 3). However, as we now have access to an orthogonal source of reference genomes (as described in Response 1.3), we have been able to construct sequence identity dotplots, and calculate the L50 and L95 for our assemblies. We believe for the GridION data there are 4 species for which we yield a single contig assembly (Table 6) and have updated our manuscript with this figure. The contiguity plot suggests that for at least one assembly condition, we recover *L. fermentum* in a single contig, but we do not have the corresponding PacBio reference. We thank the reviewer for suggesting this statement be clarified with more evidence, we believe Table 6 addresses the issues raised.

**R1.6.** In abstract "even and odd communities" should be ' evenly-distributed and log-distributed communities for clarity (this term is otherwise unclear to casual reader of abstract)

**Response 1.6**: The incorrect reference to the “odd community” has been corrected.

---

## Reviewer 2

**R2.1.** Line 28-29: Would suggest that the authors provide the citation (15) without the statement in parenthesis or revised version of statement in parenthesis.

**Response 2.1**: We thank the reviewer for this comment, the manuscript has been revised accordingly.

**R2.2.** "DNA extraction protocol" section: The last few lines were a little bit unclear. For instance: "45 ul (Even) and 225ul (Log) of the supernatant retained earlier…" It was a bit confusing. Possibly because the line "The standard was spun…before removing the supernatant and retaining." seems incomplete. I would suggest that the authors consider posting the entire protocol on protocols.io - as is quite possible that other groups may want to reproduce the sequencing step for these mock community standards. This would be particularly helpful as the authors suggest that the protocol was modified to increase fragment length.

**Response 2.2**: We agree the manuscript could be clearer in this section and have clarified the points raised. We thank the reviewer for suggesting we post the DNA extraction methods on protocols.io, we have made the method available via dx.doi.org/10.17504/protocols.io.x9tfr6n.

**R2.3.** "Illumina sequencing" section: Suggest that the authors improve clarity in this section by re-structuring this paragraph. For instance, early in paragraph it is stated that the pooled library was sequenced on four lanes on Illumina HiSeq 1500, but later stated that the even community was sequenced on a MiSeq.

**Response 2.3**: The Illumina sequencing section has been edited for clarity as suggested. The phrase “pooled library” has been changed to “multiplexed isolates” to distinguish the simultaneous sequencing of the individual isolates as different from the shotgun sequencing of the even community itself.

**R2.4.** “Table 2, Figure 3a”. - please fix this to Figure 1a.

**Response 2.4**: Thank you for noticing this incorrect reference, it has been fixed.

**R2.5.** Figure 1B: The x-axis is "accuracy" while in this section Figure 1b is referred to as providing "quality scores". Please replace "quality scores" with "accuracy" for consistency.

**Response 2.5**: The paragraph has been updated for consistency.

**R2.6.** Figure 1C: Please provide a legend mapping colors to "even" and "log". I realize this information is in Figure 1B, but would be helpful for the reader. Finally, there is no significant trend in sequencing speed over time. Considering this, would be easier to remove the Time component and just have a single panel with the GridION and PromethION sequencing speed for both even and log community in the same panel. It would make it easier to compare the different in sequencing speeds visually.

**Response 2.6**: We thank the reviewer for this useful comment. Figure 1c has been replaced with a simpler density plot showing the distribution of sequencing speeds for the four runs, and we have also added a suitable colour-coded legend for the reader.

**R2.7.** Table 5 is mentioned before Tables 3 and 4. Please correct this.

**Response 2.7**: Table 5 has been moved to earlier in the manuscript and is now referred to as Table 3.

**R2.8.** For Figure 2, consider also providing figure for the even community.

**Response 2.8**: We appreciate this comment but feel that the information for the even community is better represented by the numerical values provided in Table 4.

**R2.9.** Further, it would be helpful to get clarity on where the data for Figure 2 is coming from. Is this from mapping of long-reads to mock community draft (I think so) or from the kraken analyses.

**Response 2.9**: Our reviewer is correct that the data was from the mapping of long-reads to the Illumina mock community draft, and we agree that the caption could be clearer. The figure legend has been clarified to explicitly state the mapping method used.

**R2.10.** It is unclear how the genome completeness was estimated.

**Response 2.10**: The latest version of the manuscript now formally estimates genome completeness using measurements of L50/L95 against the reference genome, and CheckM completeness estimates (as described more fully in Response 1.3). The Methods section has been updated accordingly.

**R2.11.** The consensus accuracy data is provided for all assemblies combined. Would be helpful if there was some discussion on accuracy of assemblies as a function of wtdgb2 parameters tested. There is some discussion of this in the "Discussion section", but would be helpful if this was laid out clearly in the results, with an additional appropriate figure/table.

**Response 2.11**: We agree that the manuscript would benefit from a presentation of assembly accuracy. As described in Response 1.3, with the availability of a recently released orthogonal data set, we can now estimate accuracy and genome completeness. We believe Table 6 lays out these results clearly.

---

We thank the reviewers for their insightful and constructive feedback, which has helped us improve the manuscript, and for this opportunity to respond.

Sam Nicholls & Nicholas Loman
(On behalf of the Authors)
