# Supplementary material for: Ultra-deep, long-read nanopore sequencing of mock microbial community standards
Source: Gigascience. 2019 May 15;8(5):giz043. doi: 10.1093/gigascience/giz043 (PMC6520541; doi:10.1093/gigascience/giz043)
Supplement: Response_to_Reviewer_Comments_Original_Submission.pdf [file giz043_response_to_reviewer_comments_original_submission.pdf]

Author's Response To Reviewer Comments

Close

Dear Editor,
Since our previous submission, our manuscript has been updated. In particular we have re-performed basecalling on the original nanopore data with an updated basecaller. Additionally, we have made comparisons between our long-read assemblies and some recently released PacBio assemblies of individuals from the same mock community. We believe this addresses the primary concern of our reviewers.

We provide detailed responses to the specific comments of our reviewers below.

**

Reviewer 1
R1.1. It seems that species assignment was done in two ways, one by using Kraken on the contigs (with a database of many bacterial/viral/fungal genomes) ; and also by mapping the reads directly to the illumina assemblies of the isolates in the mixture. It would be useful to be clearer in the results which approach was used in reporting the results.
E.g. the sentence "We identify the presence of all 10 microbial species in the community, for both even and log samples, in expected proportions (Figure 2)." presumably relates to the analysis just mapping to the draft illumina assemblies?

Response 1.1: We agree that the manuscript could be clearer in this regard. The read-level analysis identified taxa by mapping to a combined Illumina assembly of all the isolates. The assembly-level analysis used kraken2 to assign contigs to species. We have clarified our Methods; in particular, the "Bioinformatics" subsection of the Methods has been elevated to a section of its own, allowing us to add suitable subheadings to better delimit our computational methods.

R1.2. Also, It seems a little surprising that there were no false positive identification of species not present in the mixture. Is this because this analysis is based on mapping to the draft illumina isolate assemblies only (see previous comment). Or, if based on kraken assignment of contigs, perhaps repetitive and/or short contigs were filtered out?

Response 1.2: The reviewer is correct that this is because analysis is based on mapping to the Illumina assemblies only with minimap2. If kraken2 had been used on individual reads, some level of misassignment would be expected.

R1.3. Could the authors present more statistics on the quality of the nanopore metagenomic assemblies, including the presence of misassemblies, any chimeric contigs, checkM completeness results; indel errors, mismatch errors, etc.

Response 1.3: We thank the reviewer for this helpful suggestion. In our initial manuscript this was difficult because of the absence of high quality whole-genome assemblies that would permit orthogonal assessment of assembly correctness. However, in the past weeks, PacBio assemblies of 7 out of 8 bacteria present in the Zymo community (excluding Lactobacillus fermentum) have been made available in McIntyre et al. Nature Communications 2019. We were able to use these genomes to assess correctness of our assemblies. Although PacBio draft assemblies are available also for S. cerevisiae (but not C. neoformans), we did not include it in the comparison because they were still heavily fragmented. We have updated the manuscript with extensive new results (Table 6). This table demonstrates dotplots of our assembled metagenomic contigs to these new PacBio references, permitting assessment of misassemblies. Additionally, Table 6 demonstrates the CheckM completeness score for the 7 bacteria that have a corresponding PacBio assembly. Because the CheckM score is heavily contingent on consensus-level sequence identity and reflects presence of indel errors, we have included completeness

results for common polishing pipelines: Racon (x2), plus Medaka, plus Illumina polishing with Pilon (x2).

R1.4. Also, can the authors confirm that the assemblies were done on the full nanopore dataset (rather than, for example, on each isolate separately after mapping the reads to each isolate draft illumina assembly).

Response 1.4: We can confirm that the assemblies are constructed from nanopore sequencing of the entire community (using 100% of GridION data, and a random 25% subsampling of PromethION sequence data). No read binning or pre-assignment steps were employed. We hope that our changes described in Response 1.1 have clarified the manuscript for readers.

R1.5. The authors write: "For the even community, using wtdgb2 with varying parameter choices, we were able to assemble seven of the bacteria into single contigs." , however this does not seem to be borne out by figure 3? I could only see 4 species with at least one single contig assembly. Perhaps the authors could spell out which species have a single contig assembly?

Response 1.5: Our assessments were initially conducted by inspecting the contiguity visualisation (Figure 3). However, as we now have access to an orthogonal source of reference genomes (as described in Response 1.3), we have been able to construct sequence identity dotplots, and calculate the L50 and L95 for our assemblies. We believe for the GridION data there are 4 species for which we yield a single contig assembly (Table 6) and have updated our manuscript with this figure. The contiguity plot suggests that for at least one assembly condition, we recover L. fermentum in a single contig, but we do not have the corresponding PacBio reference. We thank the reviewer for suggesting this statement be clarified with more evidence, we believe Table 6 addresses the issues raised.

R1.6. In abstract "even and odd communities" should be ' evenly-distributed and log-distributed communities for clarity (this term is otherwise unclear to casual reader of abstract)

Response 1.6: The incorrect reference to the “odd community” has been corrected.

**

Reviewer 2
R2.1. Line 28-29: Would suggest that the authors provide the citation (15) without the statement in parenthesis or revised version of statement in parenthesis.

Response 2.1: We thank the reviewer for this comment, the manuscript has been revised accordingly.

R2.2. "DNA extraction protocol" section: The last few lines were a little bit unclear. For instance: "45 ul (Even) and 225ul (Log) of the supernatant retained earlier…" It was a bit confusing. Possibly because the line "The standard was spun…before removing the supernatant and retaining." seems incomplete. I would suggest that the authors consider posting the entire protocol on protocols.io - as is quite possible that other groups may want to reproduce the sequencing step for these mock community standards. This would be particularly helpful as the authors suggest that the protocol was modified to increase fragment length.

Response 2.2: We agree the manuscript could be clearer in this section and have clarified the points raised.
We thank the reviewer for suggesting we post the DNA extraction methods on protocols.io, we have made the method available via dx.doi.org/10.17504/protocols.io.x9tfr6n.

R2.3. "Illumina sequencing" section: Suggest that the authors improve clarity in this section by re-structuring this paragraph. For instance, early in paragraph it is stated that the pooled library was sequenced on four lanes on Illumina HiSeq 1500, but later stated that the even community was sequenced on a MiSeq.

Response 2.3: The Illumina sequencing section has been edited for clarity as suggested. The phrase

“pooled library” has been changed to “multiplexed isolates” to distinguish the simultaneous sequencing of the individual isolates as different from the shotgun sequencing of the even community itself.

R2.4. “Table 2, Figure 3a”. - please fix this to Figure 1a.

Response 2.4: Thank you for noticing this incorrect reference, it has been fixed.

R2.5. Figure 1B: The x-axis is "accuracy" while in this section Figure 1b is referred to as providing "quality scores". Please replace "quality scores" with "accuracy" for consistency.

Response 2.5: The paragraph has been updated for consistency.

R2.6. Figure 1C: Please provide a legend mapping colors to "even" and "log". I realize this information is in Figure 1B, but would be helpful for the reader. Finally, there is no significant trend in sequencing speed over time. Considering this, would be easier to remove the Time component and just have a single panel with the GridION and PromethION sequencing speed for both even and log community in the same panel. It would make it easier to compare the different in sequencing speeds visually.

Response 2.6: We thank the reviewer for this useful comment. Figure 1c has been replaced with a simpler density plot showing the distribution of sequencing speeds for the four runs, and we have also added a suitable colour-coded legend for the reader.

R2.7. Table 5 is mentioned before Tables 3 and 4. Please correct this.

Response 2.7: Table 5 has been moved to earlier in the manuscript and is now referred to as Table 3.

R2.8. For Figure 2, consider also providing figure for the even community.

Response 2.8: We appreciate this comment but feel that the information for the even community is better represented by the numerical values provided in Table 4.

R2.9. Further, it would be helpful to get clarity on where the data for Figure 2 is coming from. Is this from mapping of long-reads to mock community draft (I think so) or from the kraken analyses.

Response 2.9: Our reviewer is correct that the data was from the mapping of long-reads to the Illumina mock community draft, and we agree that the caption could be clearer. The figure legend has been clarified to explicitly state the mapping method used.

R2.10. It is unclear how the genome completeness was estimated.

Response 2.10: The latest version of the manuscript now formally estimates genome completeness using measurements of L50/L95 against the reference genome, and CheckM completeness estimates (as described more fully in Response 1.3). The Methods section has been updated accordingly.

R2.11. The consensus accuracy data is provided for all assemblies combined. Would be helpful if there was some discussion on accuracy of assemblies as a function of wtdgb2 parameters tested. There is some discussion of this in the "Discussion section", but would be helpful if this was laid out clearly in the results, with an additional appropriate figure/table.

Response 2.11: We agree that the manuscript would benefit from a presentation of assembly accuracy. As described in Response 1.3, with the availability of a recently released orthogonal data set, we can now estimate accuracy and genome completeness. We believe Table 6 lays out these results clearly.

**

We thank the reviewers for their insightful and constructive feedback, which has helped us improve the manuscript, and for this opportunity to respond.

Sam Nicholls & Nicholas Loman
(On behalf of the Authors)

Close
