# Supplementary material for: Ultra-deep, long-read nanopore sequencing of mock microbial community standards
Source: Gigascience. 2019 May 15;8(5):giz043. doi: 10.1093/gigascience/giz043 (PMC6520541; doi:10.1093/gigascience/giz043)
Supplement: Response_to_Reviewer_Comments_Revision_1.pdf [file giz043_response_to_reviewer_comments_revision_1.pdf]

Author's Response To Reviewer Comments

Close

See cover letter

Close
