# Supplementary material for: Ultra-deep, long-read nanopore sequencing of mock microbial community standards
Source: Gigascience. 2019 May 15;8(5):giz043. doi: 10.1093/gigascience/giz043 (PMC6520541; doi:10.1093/gigascience/giz043)
Supplement: Reviewer_1_Report_Original_Submission -- Lachlan Coin — 12/29/2018 Reviewed [file giz043_reviewer_1_report_original_submission.pdf]

Reviewer Report

Title: Ultra-deep, long-read nanopore sequencing of mock microbial community standards

Version: Original Submission **Date:** 12/29/2018

Reviewer name: Lachlan Coin

Reviewer Comments to Author:

This is a great data resource, and will be invaluable to the community for testing/developing approaches for metagenome assembly. The aims are well described. Aside from a few queries I have below, the conclusions are largely supported by data shown; the manuscript is well written, and there are no statistical tests presented.
Major comments:
It seems that species assignment was done in two ways, one by using Kraken on the contigs (with a database of many bacterial/viral/fungal genomes) ; and also by mapping the reads directly to the illumina assemblies of the isolates in the mixture. It would be useful to be clearer in the results which approach was used in reporting the results. E.g. the sentence " We identify the presence of all 10 microbial species in the community, for both even and log samples, in expected proportions(Figure 2). " presumably relates to the analysis just mapping to the draft illumina assemblies?
- Also, It seems a little surprising that there were no false positive identification of species not present in the mixture. Is this because this analysis is based on mapping to the draft illumina isolate assemblies only (see previous comment). Or, if based on kraken assignment of contigs, perhaps repetitive and/or short contigs were filtered out?
- Could the authors present more statistics on the quality of the nanopore metagenomic assemblies, including the presence of misassemblies, any chimeric contigs, checkM completeness results; indel errors, mismatch errors, etc.
- Also, can the authors confirm that the assemblies were done on the full nanopore dataset (rather than, for example, on each isolate separately after mapping the reads to each isolate draft illumina assembly).
The authors write : " For the even community, using wtdgb2 with varying parameter
choices, we were able to assemble seven of the bacteria into
single contigs." , however this does not seem to be borne out by figure 3? I could only see 4 species with at least one single contig assembly. Perhaps the authors could spell out which species have a single contig assembly?
Minor Comments:
- In abstract "even and odd communities" should be ' evenly-distributed and log-distributed communities for clarity (this term is otherwise unclear to casual reader of abstract)

Level of Interest

Please indicate how interesting you found the manuscript: Choose an item.

Quality of Written English

Please indicate the quality of language in the manuscript: Choose an item.

Declaration of Competing Interests

Please complete a declaration of competing interests, considering the following questions:

- Have you in the past five years received reimbursements, fees, funding, or salary from an organisation that may in any way gain or lose financially from the publication of this manuscript, either now or in the future?
- Do you hold any stocks or shares in an organisation that may in any way gain or lose financially from the publication of this manuscript, either now or in the future?
- Do you hold or are you currently applying for any patents relating to the content of the manuscript?
- Have you received reimbursements, fees, funding, or salary from an organization that holds or has applied for patents relating to the content of the manuscript?
- Do you have any other financial competing interests?
- Do you have any non-financial competing interests in relation to this paper?

If you can answer no to all of the above, write 'I declare that I have no competing interests' below. If your reply is yes to any, please give details below.

I have received free registration and travel expense reimbursement to a nanopore community meeting. I have also participated in the Oxford Nanopore Technologies early access program. Other than this I have no competing interests.

I agree to the open peer review policy of the journal. I understand that my name will be included on my report to the authors and, if the manuscript is accepted for publication, my named report including any attachments I upload will be posted on the website along with the authors' responses. I agree for my report to be made available under an Open Access Creative Commons CC-BY license (http://creativecommons.org/licenses/by/4.0/). I understand that any comments which I do not wish to be included in my named report can be included as confidential comments to the editors, which will not be published.

I agree to the open peer review policy of the journal

To further support our reviewers, we have joined with Publons, where you can gain additional credit to further highlight your hard work (see: https://publons.com/journal/530/gigascience). On publication of this paper, your review will be automatically added to Publons, you can then choose whether or not to claim your Publons credit. I understand this statement.

Yes Choose an item.
