# Supplementary material for: Ultra-deep, long-read nanopore sequencing of mock microbial community standards
Source: Gigascience. 2019 May 15;8(5):giz043. doi: 10.1093/gigascience/giz043 (PMC6520541; doi:10.1093/gigascience/giz043)
Supplement: Reviewer_2_Report_Original_Submission -- Ameet Pinto, PhD — 1/3/2019 Reviewed [file giz043_reviewer_2_report_original_submission.pdf]

Reviewer Report

Title: Ultra-deep, long-read nanopore sequencing of mock microbial community standards

Version: Original Submission **Date:** 1/3/2019

Reviewer name: Ameet Pinto, PhD

Reviewer Comments to Author:

The manuscript provides long-read mock community datasets from GridION and PromethION sequencing platforms along with draft genomes of mock community organisms sequenced on the Illumina Platform. The entire dataset is available for reuse by the research community and this is an extremely valuable resource that the authors have made available. While there are some analyses of the data included in the current manuscript, it is largely limited to summary statistics (which seems appropriate for a Data Note type manuscript) and some analyses of interest to the field (e.g., de novo metagenome assembly). It would have been helpful to have a more detailed evaluation of the de novo assembly and parameter optimization, but this may have been outside the scope of a Data Note type manuscript. I have some minor comments below to improve clarity of the manuscript.

Minor comments:

1. Line 28-29: Would suggest that the authors provide the citation (15) without the statement in parenthesis or revised version of statement in parenthesis.

"DNA extraction protocol" section

2. The last few lines were a little bit unclear. For instance: "45 ul (Even) and 225ul (Log) of the supernatant retained earlier…" It was a bit confusing. Possibly because the line "The standard was spun…before removing the supernatant and retaining." seems incomplete. I would suggest that the authors consider posting the entire protocol on protocols.io - as is quite possible that other groups may want to reproduce the sequencing step for these mock community standards. This would be particularly helpful as the authors suggest that the protocol was modified to increase fragment length.

"Illumina sequencing" section:

3. Suggest that the authors improve clarity in this section by re-structuring this paragraph. For instance, early in paragraph it is stated that the pooled library was sequenced on four lanes on Illumina HiSeq 1500, but later stated that the even community was sequenced on a MiSeq.

"Nanopore sequencing metrics" in results:

4. Table 2, Figure 3a. - please fix this to Figure 1a.

5. Figure 1B: The x-axis is "accuracy" while in this section Figure 1b is referred to as providing "quality scores". Please replace "quality scores" with "accuracy" for consistency.

6. Figure 1C: Please provide a legend mapping colors to "even" and "log". I realize this information is in Figure 1B, but would be helpful for the reader. Finally, there is no significant trend in sequencing speed over time. Considering this, would be easier to remove the Time component and just have a single panel with the GridION and PromethION sequencing speed for both even and log community in the same panel. It would make it easier to compare the different in sequencing speeds visually.

"Illumina sequencing metrics" in results:

7. Table 5 is mentioned before Tables 3 and 4. Please correct this.
"Nanopore mapping statistics" in results:
8. For Figure 2, consider also providing figure for the even community.
9. Further, it would be helpful to get clarity on where the data for Figure 2 is coming from. Is this from mapping of long-reads to mock community draft (I think so) or from the kraken analyses.
"Nanopore metagenome assemblies" in results:
1. It is unclear how the genome completeness was estimated.
2. The consensus accuracy data is provided for all assemblies combined. Would be helpful if there was some discussion on accuracy of assemblies as a function of wtdgb2 parameters tested. There is some discussion of this in the "Discussion section", but would be helpful if this was laid out clearly in the results, with an additional appropriate figure/table.

Level of Interest

Please indicate how interesting you found the manuscript: Choose an item.

Quality of Written English

Please indicate the quality of language in the manuscript: Choose an item.

Declaration of Competing Interests

Please complete a declaration of competing interests, considering the following questions:

- Have you in the past five years received reimbursements, fees, funding, or salary from an organisation that may in any way gain or lose financially from the publication of this manuscript, either now or in the future?
- Do you hold any stocks or shares in an organisation that may in any way gain or lose financially from the publication of this manuscript, either now or in the future?
- Do you hold or are you currently applying for any patents relating to the content of the manuscript?
- Have you received reimbursements, fees, funding, or salary from an organization that holds or has applied for patents relating to the content of the manuscript?
- Do you have any other financial competing interests?
- Do you have any non-financial competing interests in relation to this paper?

If you can answer no to all of the above, write 'I declare that I have no competing interests' below. If your reply is yes to any, please give details below.

I declare that I have no competing interests

I agree to the open peer review policy of the journal. I understand that my name will be included on my report to the authors and, if the manuscript is accepted for publication, my named report including any attachments I upload will be posted on the website along with the authors' responses. I agree for my report to be made available under an Open Access Creative Commons CC-BY license (http://creativecommons.org/licenses/by/4.0/). I understand that any comments which I do not wish to be included in my named report can be included as confidential comments to the editors, which will not be published.

I agree to the open peer review policy of the journal

To further support our reviewers, we have joined with Publons, where you can gain additional credit to further highlight your hard work (see: https://publons.com/journal/530/gigascience). On publication of this paper, your review will be automatically added to Publons, you can then choose whether or not to claim your Publons credit. I understand this statement.

Yes Choose an item.
